# Comprehensive Characterization of the Immune Microenvironment Based on Nested Resampling Machine Learning Framework Identifies TRAF3 Interacting Protein 3 as a Promising Regulator to Improve the Resistance to Immunotherapy in Glioma

**DOI:** 10.1002/advs.202502271

**Published:** 2025-07-26

**Authors:** Yanbo Yang, Fei Wang, Yulian Zhang, Run Huang, Chuanpeng Zhang, Lu Zhao, Hanhan Dang, Xinyu Tao, Yue Lu, Dengfeng Lu, Yunsheng Zhang, Kun He, Jiancong Weng, Zhouqing Chen, Zhong Wang, Yanbing Yu

**Affiliations:** ^1^ Department of Neurosurgery The First Affiliated Hospital of Soochow University Suzhou Jiangsu 215006 China; ^2^ Department of Neurosurgery China‐Japan Friendship Hospital Chinese Academy of Medical Sciences & Peking Union Medical College Beijing 100000 China; ^3^ Department of Oncology Division of Pediatric Oncology Johns Hopkins School of Medicine Baltimore Maryland 21231 US; ^4^ Department of Neurosurgery China‐Japan Friendship Hospital Beijing 100000 China; ^5^ Suzhou Medical College of Soochow University Suzhou Jiangsu 215127 China; ^6^ Department of Neurosurgery Peking University China‐Japan Friendship School of Clinical Medicine Beijing 100000 China; ^7^ China‐Japan Friendship Hospital Capital Medical University Beijing 100000 China; ^8^ State Key Laboratory of Phytochemistry and Plant Resources in West China Kunming Institute of Botany Chinese Academy of Sciences Kunming Yunnan 650201 China; ^9^ Department of Neurosurgery Peking University Civil Aviation School of Clinical Medicine Beijing 100000 China

**Keywords:** glioma, machine learning, multi‐omics, PDL1, TRAF3IP3, tumor microenvironment

## Abstract

Diffuse glioma, the most prevalent and malignant intracranial tumor, presents a formidable challenge due to its immunosuppressive microenvironment, which complicates conventional therapeutic approaches. This study conducted a comprehensive prognostic meta‐analysis involving 2,968 patients with diffuse glioma and established a comprehensive machine learning framework with nested resampling of 18 machine learning algorithms, and developed the Immune Glioma Survival Signature (IGLoS). This signature, comprising CCL19, ICOSLG, IL11, PTGES, TNFAIP3, and TRAF3IP3, has been demonstrated to predict survival outcomes across a range of cancers and to correlate with tumor progression at the level of multi‐omics. It is noteworthy that the IGLoS score enables precise patient stratification for personalized cancer treatments and elucidates pivotal resistance mechanisms to immunotherapy. Furthermore, siRNA screening has underscored the critical role of TRAF3IP3 in modulating PDL1 expression and immune pathways, with implications on the ERK pathway and NFATC2 involvement. Through single‐cell analysis of published and in‐house datasets, TRAF3IP3 exhibited selective enrichment in NPC‐like and MES‐like tumor cells, and showed a dual functionality in mediating T‐Cell Exhaustion. Targeting TRAF3IP3 emerges as a promising avenue to combat immunotherapy resistance, particularly in glioma, thus paving the way for precision medicine.

## Graphical abstract

This study develops a nested resampling machine learning framework to establish the Immune Glioma Survival Signature (IGLoS), which stratifies glioma patients and predicts immunotherapy resistance. Multi‐omics analyses reveal TRAF3IP3 as a pivotal regulator of PD‐L1 expression and T‐cell exhaustion via ERK‐NFATC2 signaling. Targeting TRAF3IP3 emerges as a strategy to overcome immunosuppression, offering insights for precision therapy in glioma.

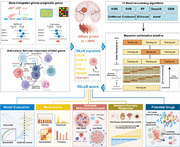

## 1 Introduction

The majority of primary malignant brain tumors in adults are of glial origin,^[^ ^1^ ^]^ of which ≈49% are glioblastomas, and thirty percent of them are diffusely infiltrating low‐grade gliomas that often progress to more aggressive, high‐grade tumors,^[^ ^2^, ^3^ ^]^ with reported median survival durations of 14–17 months^[^ ^4^ ^]^ and more than 7 years,^[^ ^5^ ^]^ respectively. Over the past years, the advent of immunotherapy has revolutionized both the clinical practice of oncology and our understanding of cancer biology.^[^ ^6^ ^]^ Unfortunately, despite remarkable success observed in other malignancies, all immunotherapies tested to date have failed to improve clinical outcomes in unselected cohorts of patients with diffuse glioma, particularly glioblastoma.^[^ ^7^, ^8^, ^9^ ^]^ Most likely, the failure of immunotherapy is attributed to the limited patient stratification methods to rationally select patients who might benefit most from immunotherapy,^[^ ^10^ ^]^ because the frequency of established biomarkers is very low in glioma.^[^ ^11^, ^12^, ^13^ ^]^ Therefore, efforts are still needed to identify effective stratification criteria and screen novel regulators that can activate the immunosuppressive microenvironment of gliomas.

Several groups have reported robust associations between tumor cell phenotypes and inflammatory features of the tumor microenvironment (TME) in gliomas,^[^ ^14^, ^15^ ^]^ which consists predominantly of microglia and macrophages (≈30%) but also includes endothelial cells, pericytes, astrocytes, lymphocytes, and neurons.^[^ ^16^ ^]^ Recent studies have suggested that non‐neoplastic cells within the TME could influence glioma cell phenotype,^[^ ^17^ ^]^ and glioma cells may also modulate the recruitment and phenotype of myeloid cells, microglia, and reactive glial cells.^[^ ^18^ ^]^ This dynamic crosstalk between gliomas and immune cells and other non‐neoplastic cells ultimately shapes and contributes to the variable inflammatory phenotypes and heterogeneity in glioma TME,^[^ ^19^ ^]^ thus resulting in increased tumor aggressiveness, migrative potential, and contributing to the immunosuppressive environment.^[^ ^20^, ^21^ ^]^ Immunotherapeutic strategies targeting glioma will need to take the landscape of TME into account and will require a more complete understanding of how the glioma‐associated immune response is regulated and how it evolves with disease progression.^[^ ^3^ ^]^

Advances in high‐throughput technologies have generated large amounts of transcriptomic gene expression data from glioma samples, including those with response information, which provide valuable resources for the identification of immunotherapy response biomarkers for patients with glioma.^[^ ^22^ ^]^ With the advent of the big data era, machine learning has been widely applied to detect important features from complex datasets, and it has been popular in clinical cancer research in recent years, being instrumental in early diagnosis, subtype identification, prognosis prediction, and so on.^[^ ^23^, ^24^, ^25^ ^]^ However, previous studies mainly pooled a small subset of available datasets, while the integrative analysis of public‐available datasets could reveal tumor–immune cell interactions and develop reliable biomarkers for predicting immunotherapy response in gliomas.

In this study, we adopted a meta‐analysis pipeline, pooling the evidence from all the available glioma datasets and conducted a prognostic meta‐analysis on 2968 diffuse patients with glioma. With 17 immune cell signatures and 15 immune function signatures, we selected six immune infiltration‐related genes, forming the Immune Glioma Survival Signature (IGLoS signature). Using a nested‐resampling approach benchmarking 18 available machine‐learning algorithms, we developed a machine learning model with the signature and further applied the IGLoS score for patient stratification and precision treatment, and validated the model with an in‐house glioma cohort. Finally, we performed the siRNA screen to further select targets whose knockdown could downregulate PDL1 expression.

## 2 Results

### 2.1 Identification of the IGLoS Signature in Glioma Cohorts

**Figure 1:**
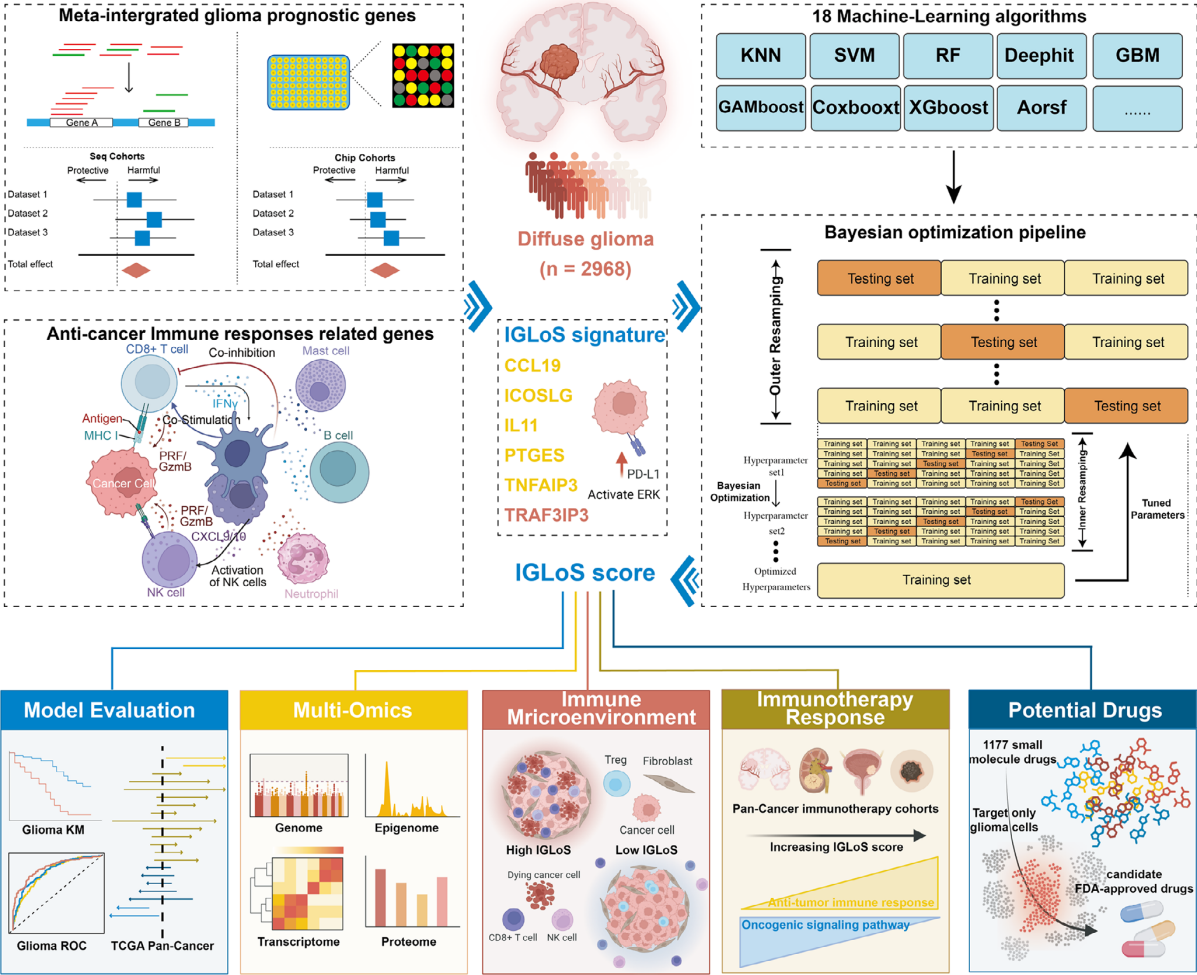
Study design.

**Figure 2:**
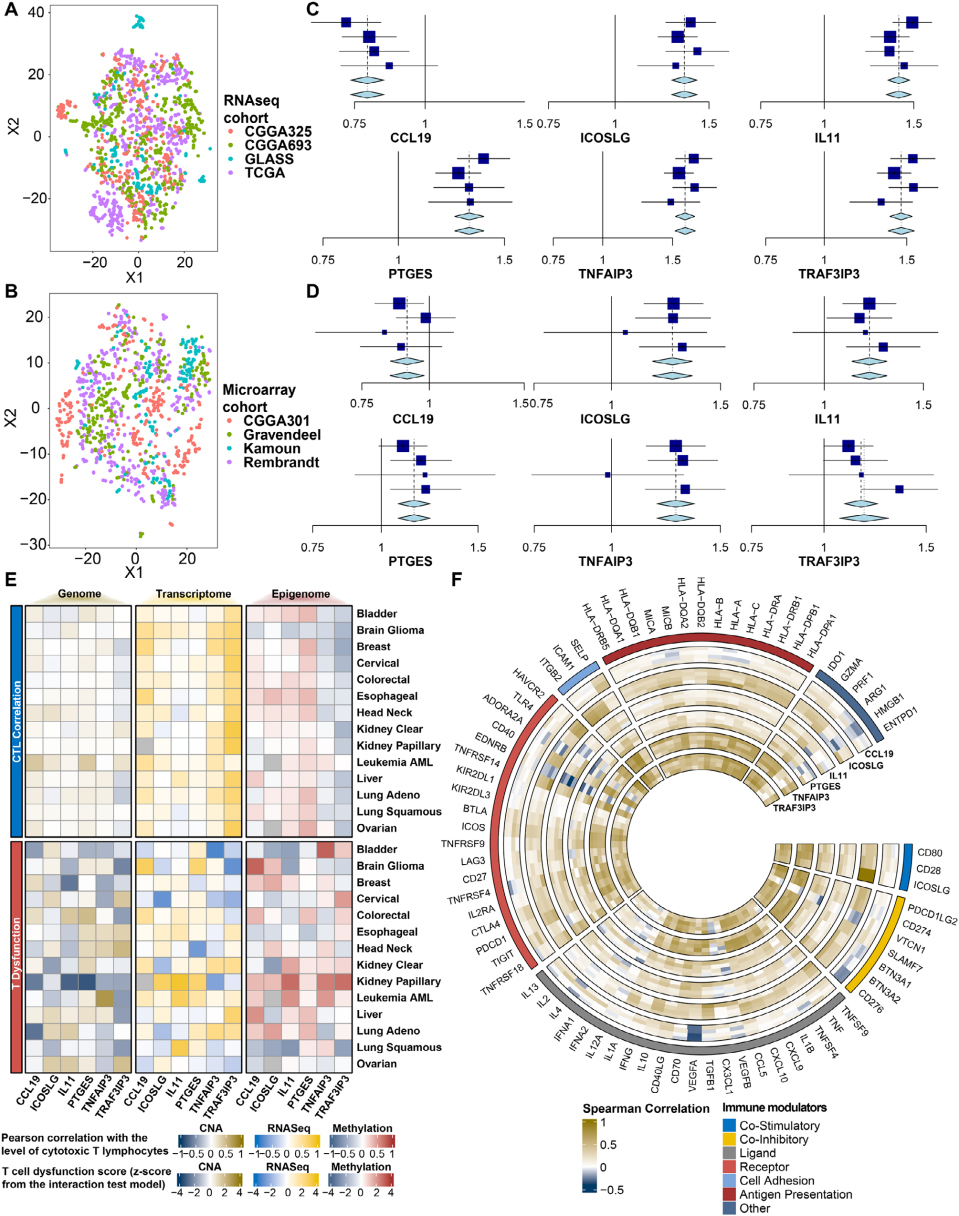
Meta‐analysis of the IGLoS signature and association with immune modulators in glioma and pan‐cancer cohorts. A) tSNE analysis of RNA‐seq cohorts and microarray cohorts of glioma, including TCGA, CGGA693, CGGA325, and GLASS. B) tSNE analysis of RNA‐seq cohorts and microarray cohorts of glioma, including Rembrandt, Gravendeel, Kamoun, and CGGA301. C) Meta‐analysis of the IGLoS signature in RNA‐seq cohorts of glioma. D) Meta‐analysis of the IGLoS signature in RNA‐seq and microarray cohorts of glioma. E) The pre‐calculated T cell dysfunction score using the interaction test model (upper half of the panel) and the correlation between the level of cytotoxic T lymphocytes and gene expression (lower half of the panel). F) Correlation analyses between the IGLoS signature and the expression of 74 key immune modulators in RNA‐seq cohorts of glioma.

The overall design of this study is displayed in **Figure** **1**. tSNE analyses of the RNA‐seq cohorts and the microarray cohorts showed that batch effects between individual cohorts were removed (**Figure** **2**A,B). We identified prognostic genes by performing the univariate Cox regression for each gene, based on which the meta‐analysis identified 3072 genes in four RNA‐seq cohorts (Table S6, Supporting Information) and 3756 genes in four microarray cohorts (Table S7, Supporting Information), respectively. A total of 371 genes were considered prognostic in both meta‐analyses of RNA‐seq cohorts and microarray cohorts, which mainly engaged in immune system‐related pathways, such as the regulation of the inflammatory response, cellular response to chemical stress, and leukocyte activation (Figure S1A, Supporting Information).

Concerning the strong correlation between immune system activity with survival in patients with glioma, we systematically explored published literature and finally identified 606 immune‐infiltration‐related genes consisting of 17 immune cell signatures and 15 immune function signatures, mainly involved in pathways related to anti‐tumor immune response, such as the overview of proinflammatory and profibrotic mediators, the regulation of leukocyte cell‐cell adhesion, and lymphocyte activation (Figure S1B, Supporting Information).

After identifying 371 prognostic genes and 606 immune infiltration‐related genes, we identified 15 immune infiltration‐related genes closely associated with glioma patient survival, which were TNFAIP3, IL11, IDO1, TRAF3IP3, IL1R1, PIK3CD, PTGES, ICOSLG, IL17RA, CADM1, SLC2A5, CCL19, TPSAB1, IL21R, and ADAT2 genes, mainly engaging in the positive regulation of cytokine production, the positive regulation of immune response, and cell activation (Figure S1C, Supporting Information). Next, we used the LASSO regression to further select six key regulators consisting of CCL19, ICOSLG, IL11, PTGES, TNFAIP3, and TRAF3IP3 (Figure 2C,D), and they were named the Immune Glioma Survival Signature (IGLoS signature).

### 2.2 Multi‐Omics Landscape of the IGLoS Signature at the Pan‐Cancer Level

At the genomic level, the waterfall plot presents 286 samples that have at least one SNV (Figure S2A, Supporting Information). TNAFIP3 and TRAF3IP3 showed SNVs in more than 40% of patients, and variations in all six genes were dominated by missense mutations. ESCA, SKCM, COAD, LUAD, and STAD had the highest deleterious mutation frequencies among 24 cancer types detected SNVs, while SNV frequencies of the IGLoS signature in GBM (10/24) and LGG (17/24) are relatively low. At the epigenetic level, the bubble plot showed that the methylation levels of the IGLoS signature were negatively correlated with survival in LGG, and the methylation level of the IGLoS signature could also predict the OS and FPS in PRAD, CESC, HNSC, KIRC, BRCA, THCA, and UVM (Figure S2B, Supporting Information). At the transcriptomic level, we found that CCL19 and TRAF3IP3 were protective factors in pan‐cancer, whereas IL11, PTGES, and TNFAIP3 were risk factors (Figure S2C, Supporting Information). Also, results for gliomas were consistent with the findings of the meta‐analysis of glioma cohorts (Figure 2C,D). The IGLoS signature also demonstrated significant differences between tumor and normal tissues in pan‐cancer, among which the expression of CCL19 was lower in most tumors, while the other five genes showed higher expression in most tumors (Figure S2D, Supporting Information).

At the immunogenomic level, the correlation between multi‐omics data and the level of cytotoxic T lymphocytes in pan‐cancer showed that the level of cytotoxic T‐lymphocyte infiltration may be more closely related to transcriptomics as well as methylation genomics than genomics. Among the six genes, multi‐omics changes in TNFAIP3 and TRAF3IP3 may be involved in regulating the level of cytotoxic T‐lymphocyte infiltration at the pan‐cancer level (top half of Figure 2E). For the T cell dysfunction score, although no discernible trend was observed, the IGLoS signature may play an important role in T cell dysfunction in the immune microenvironment of papillary renal carcinoma, glioma, and breast cancer (bottom half of Figure 2E). According to the “Gene set prioritization” module of the TIDE portal, ICOSLG was identified as the optimal target to render the TME resistant to ICI. Specifically, high ICOSLG was associated with T cell dysfunction phenotypes (Figure S2E left panel, Supporting Information), worse ICI outcome (Figure S2E second to left panel, Supporting Information), and prompting T cell exclusion (Figure S2E right panel, Supporting Information). In four RNA‐seq cohorts of glioma, the correlation between the IGLoS signature and the expression of 74 key immune modulators showed an overwhelmingly positive correlation, with TNFAIP3 and TRAF3IP3 showing the most significant association with immune modulators (Figure 2F). Interestingly, as a known positive regulator, CCL19 demonstrated relatively more negative correlations in glioma, with the most significant negative correlations with VEGFA, CD276, and HMGB1.

### 2.3 Calculation and Validation of the IGLoS Score Using the Gradient Boosting Algorithm with the Highest c‐Index

**Figure 3:**
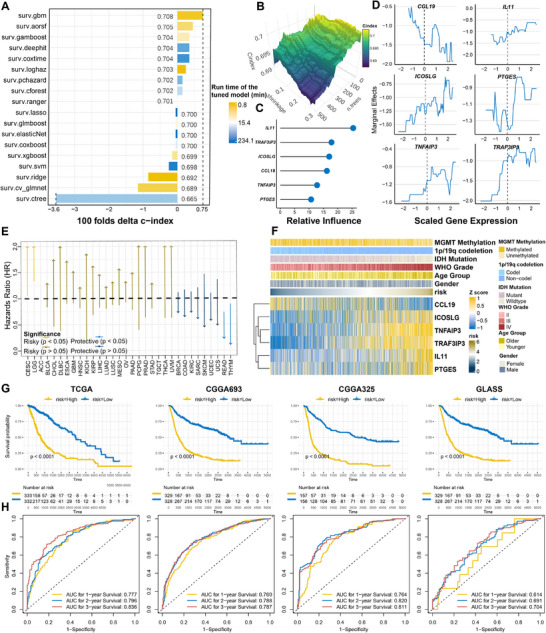
Calculation and validation of the IGLoS score using the Gradient Boosting model with the highest c‐index. A) Compared with the other 17 ML models, the Gradient Boosting model achieves the highest c‐index of 0.708 in the glioma Meta cohort and exhibits a lower runtime. B) The hyperparameter optimization process of the Gradient Boosting model. C) Relative influence is computed by the reduction of squared error attributable to each variable normalized to sum to 100. D) Marginal effect change is selected as a particular feature while averaging over or integrating out the influence of all other features E) The forest plot shows that the IGLoS score is associated with a trend toward worse prognosis in more than half of the cancers. F) The heatmap shows the distribution of clinical characteristics with the increase of the IGLoS score in the glioma Meta cohort, including sex, age, WHO grade, IDH mutation, 1*p*/19*q* codeletion, and MGMT methylation. G) The Kaplan–Meier curve demonstrates the ability of the IGLoS score to predict survival in four RNA‐seq cohorts of glioma. H) As shown on the ROC curve, the AUCs for 1‐, 2‐, and 3‐year survival show the ability of the IGLoS score to predict survival in four RNA‐seq cohorts of glioma.

Compared to the other 17 finely tuned ML models (**Figure** **3**A), the tuned Gradient Boosting model (Figure 3B) showed the highest c‐index of 0.708 for the glioma Meta‐cohort, as well as having a lower runtime, and was therefore selected for calculating the IGLoS score in four RNA‐seq cohorts of glioma, TCGA pan‐cancer cohorts, and immunotherapy cohorts (Table S8, Supporting Information). IL11 was considered the most important gene (Figure 3C,D). In TCGA pan‐cancer cohorts, the IGLoS score was associated with a worse prognosis in LGG and CESC and a better prognosis in READ and THYM. Although the IGLoS score demonstrated prognostic predictive power in only four cancers, it was associated with a trend toward worse prognosis in more than half of the cancers (Figure 3E). The reason that the IGLoS score indicated a better prognosis in some cancers might be that the expression of the IGLoS signature was dysregulated in this part of the malignancy in the opposite direction from other cancers (Figure S2D, Supporting Information).

To explore the association between the IGLoS score and clinical characteristics including sex, age, WHO grade, IDH mutation, 1*p*/19*q* codeletion, and MGMT methylation, we plotted a heatmap (Figure 3F) to show the distribution of clinical characteristics and the expression of IGLoS signature in order of the IGLoS scores from lowest to highest. Higher IGLoS scores seemed to predict higher WHO grade, IDH wild type, 1*p*/19*q* co‐deletion, and MGMT unmethylation (Table S9, Supporting Information). In addition, the Kaplan‐Meier curve and ROC curve of four RNA‐seq cohorts indicated that the IGLoS score has a powerful ability to predict the survival of patients with glioma (Figure 3G,H). Previous research has indicated that patients with IDH wild‐type GBM usually have a poor prognosis compared to other glioma subtypes.^[^ ^26^ ^]^ Therefore, we further verified the prognostic stratification ability of the IGLoS signature in IDH wild‐type GBM patients. The Kaplan‐Meier curve revealed the possible ability of the IGLoS score to predict survival in four RNA‐seq cohorts of IDH wild‐type GBM (Figure S3A, Supporting Information). The following meta‐analysis in four RNA‐seq cohorts of IDH wild‐type GBM showed that TRAF3IP3 might act as a prognostic indicator for IDH wild‐type GBM patients (Figure S3B, Supporting Information). In the Gusu in‐house dataset consisting of 24 patients with glioma, TRAF3IP3, TNFAIP3, ICOSLG, IL11, and PTGES, showed a trend of increasing expression with increasing tumor grade, whereas CCL19 was not (Figure S3C, Supporting Information).

Furthermore, we originally intended to use a trained machine learning model to predict patient prognosis using expression matrices. However, due to the limited follow‐up and small number of recorded deaths (Table S5, Supporting Information), the survival analysis currently lacks the statistical power to produce significant results. We will continue to monitor patients and extend follow‐up, and we expect the prognostic utility of the IGLoS score to be validated as additional data become available.

### 2.4 The IGLoS Score Correlates with the Development of Glioma at the Multi‐Omics Level

At the genomic level, a positive correlation between the IGLoS score and tumor mutation burden was observed (Figure S4B, Supporting Information). Comprehensive mutation information in each sample of the low‐IGLoS and high‐IGLoS groups was presented in waterfall plots. The horizontal histogram showed the top 10 mutated genes in gliomas, including TP53, IDH1, ATRX, TTN, PTEN, EGFR, MUC16, NF1, RYR2, PIK3CA, LRP2, FLG, PKHD1, PCLO, and OBSCN, in the high‐IGLoS group (Figure S4A, Supporting Information), while IDH1, TP53, ATRX, CIC, TTN, FUBP1, PIK3CA, MUC16, NOTCH1, IDH2, SMARCA4, and PIK3R1 in the low‐IGLoS group (Figure S4C, Supporting Information).

At the epigenetic level, a total of 39 differentially methylated sites were identified between the high‐IGLoS and low‐IGLoS groups in TCGA (Table S10, Supporting Information), which were distributed at different positions in the IGLoS signature (Figure S4D, Supporting Information), and 31 of 39 sites were identified as protective factors by the univariate Cox regression (Figure S4E, Supporting Information). The differential methylation probe signal diminishes as the IGLoS Score increases (Figure S4F, Supporting Information), which is consistent with the high expression of the IGLoS signature (in the exception of CCL19) in gliomas, as the methylation of genes is often thought to silence gene expression.

At the transcriptomic level, we performed GSEA and KEGG enrichment analyses of differential genes between the high‐IGLoS and low‐IGLoS groups in TCGA (Figure S4G–I, Supporting Information) and CGGA693 (Figure S4J–L, Supporting Information), and the results showed that the differential genes were mainly involved in immune response‐related pathways (such as adaptive immune response and cytokine‐cytokine receptor interactions) and nervous system‐related pathways (such as synaptic activity and neuroactive ligand‐receptor interaction).

### 2.5 IGLoS Score Reveals an Active Cross‐Talk Between Immune Cells and Glioma Cells

**Figure 4:**
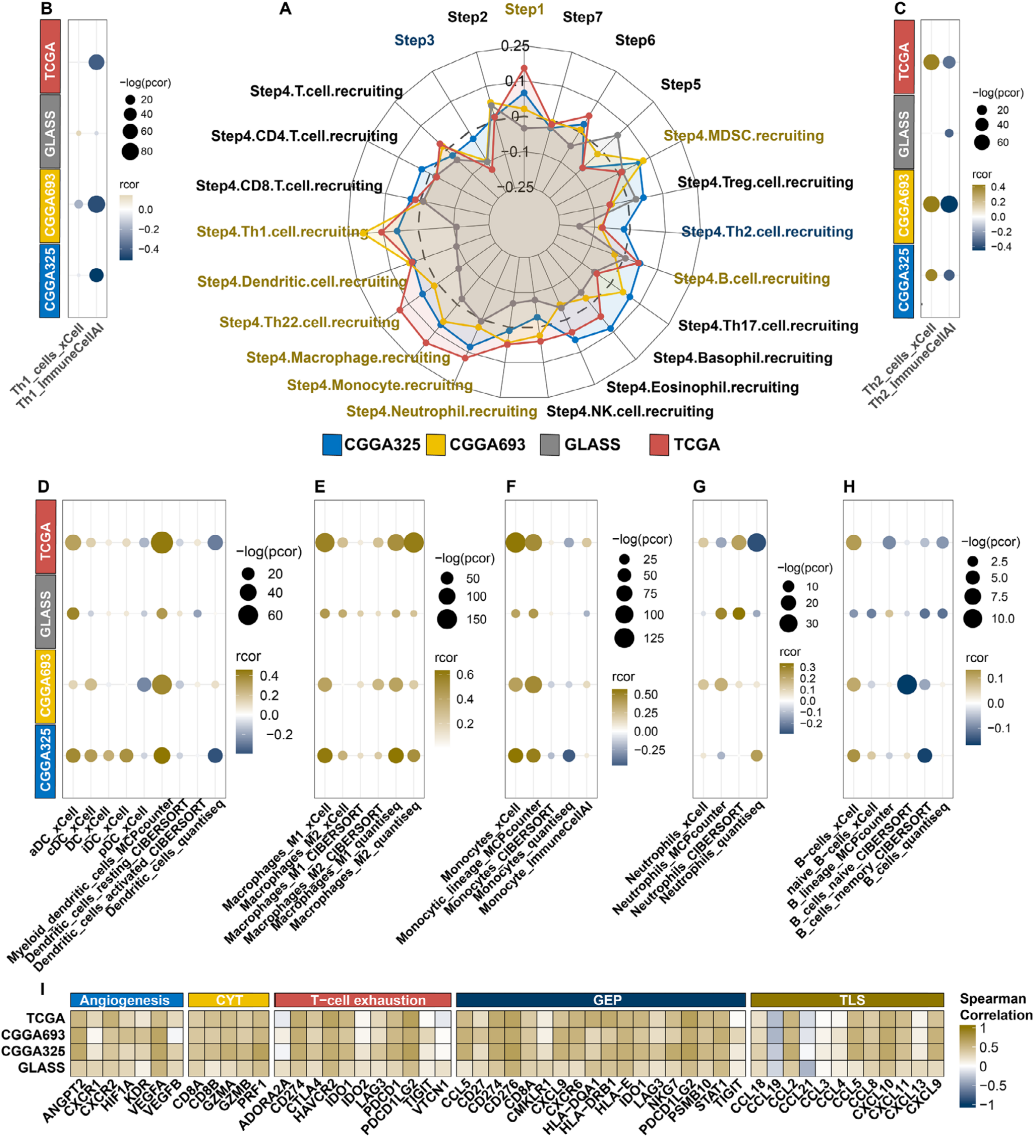
Correlation between IGLoS scores, immune cell infiltration, and key pathways in cancer immunology in glioma. A) The bubble plot shows the infiltration level of Th1 cells in patients with glioma. B) The radar plot shows the correlation between IGLoS scores and steps of antitumor immunity in four RNA‐seq cohorts of glioma. C–H) The bubble plots show the level of immune cell infiltration in patients with glioma, including Th2 cells, DCs, macrophages, monocytes, neutrophils, and B cells. I) Correlation between IGLoS scores and key pathways in cancer immunology, including angiogenesis, CYT, T‐cell exhaustion, GEP, and TLS.

The anti‐cancer immune response can be conceptualized as a series of stepwise events referred to as the Cancer‐Immunity Cycle.^[^ ^27^ ^]^ Although the correlation between IGLoS scores and the activity of each step of antitumor immunity was generally modest, nine of these steps were positively correlated with the IGLoS score in at least three glioma cohorts, whereas two steps were negatively correlated with the IGLoS score in at least three glioma cohorts (**Figure** **4**A). To further explore the level of infiltration of various immune cells during these steps, we assessed the level of immune infiltration based on gene expression data from patients with glioma using multiple algorithms (Figure 4B–H). We found that as the IGLoS score increased, there was a corresponding increase in macrophages and neutrophils and a corresponding decrease in B cells in the TME of patients with glioma. At the pan‐cancer level, IGLoS scores were generally positively correlated with the level of dendritic cell, macrophage, monocyte, and neutrophil infiltration (Figure S5A, Supporting Information). Interestingly, the negative correlation of B cells with the IGLoS scores appeared to occur only in gliomas, suggesting that B cells may receive specific regulation in gliomas.

We collected signatures of some key pathways in cancer immunology, including angiogenesis, CYT, GEP, T‐cell exhaustion, and TLS, and found that, except for CCL19 and CCL21 in the TLS pathway, which were negatively correlated with the IGLoS scores, the vast majority of genes in the other pathways were positively correlated with the IGLoS scores, with the most significant correlation being with GEP (Figure 4I). T cell compartment and its regulation were the most significant conceptual alteration in anti‐cancer immune response.^[^ ^6^ ^]^ We analyzed differences in the levels of CD4+ and CD8+ T‐cell infiltration of each subset between the high‐IGLoS and low‐IGLoS groups of patients with glioma and found that patients in the high‐IGLoS group had less CD4+ T cells (Figure S5B, Supporting Information) and more CD8+ T cells (Figure S5C, Supporting Information). Notably, in TCGA and CGGA693, the high‐IGLoS group was found to be associated with more exhausted CD8+ T cells.

### 2.6 The IGLoS Score is a Potent Predictor of ICI Response in Pan‐Cancer Immunotherapy Cohorts

**Figure 5:**
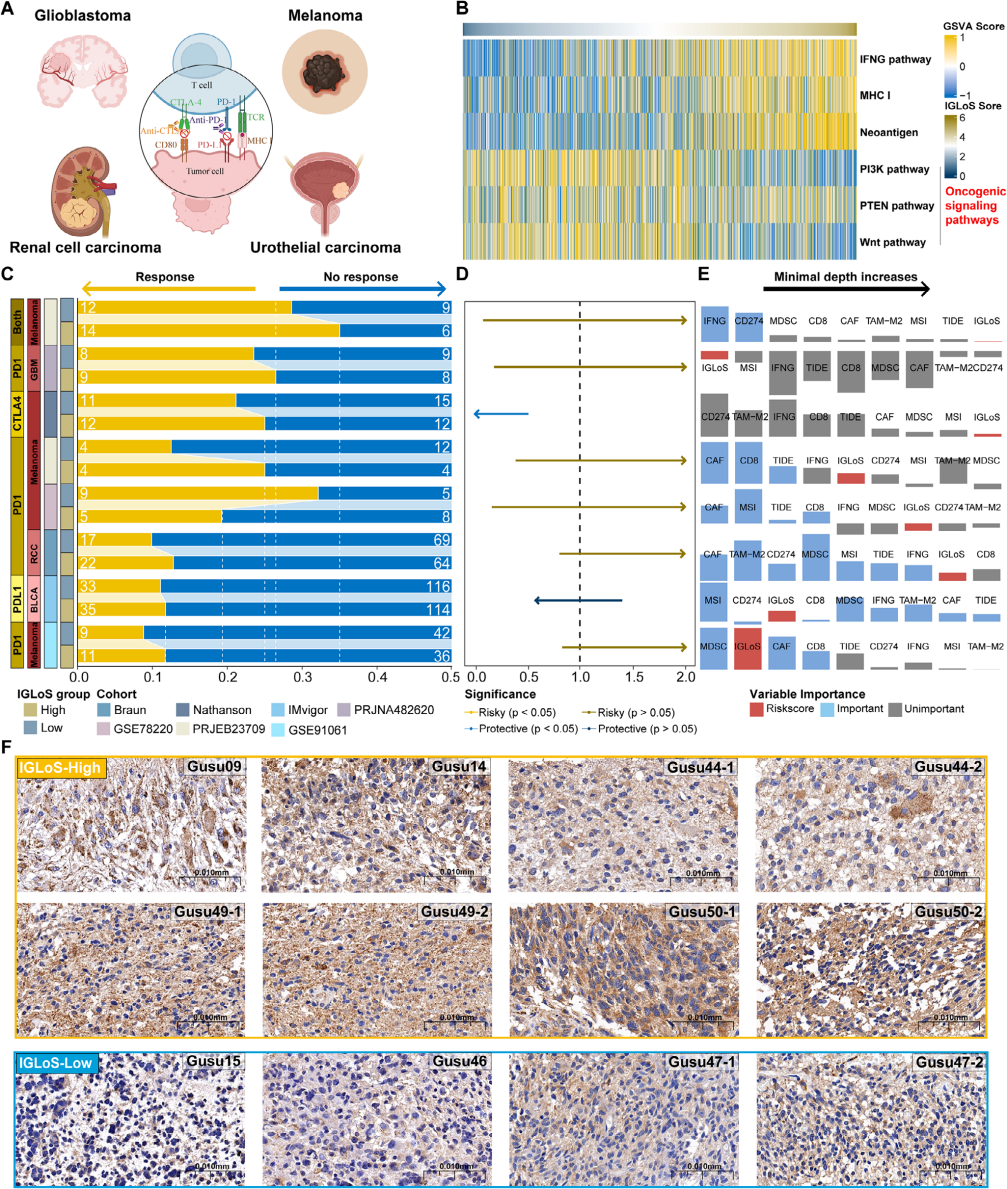
The value of the IGLoS score in predicting the response to ICI in pan‐cancer immunotherapy cohorts. A) Pan‐cancer immunotherapy cohorts with survival information were accessed from the TIGER portal. B) Correlation between IGLoS scores and the GSVA scores of tumor cells' intrinsic pathways causing resistance to ICI, including the IFNG signature, the MHC I signature, the neoantigen signature, the PI3K pathway, the PTEN pathway, and the Wnt pathway. C) The bar plots show that the IGLoS score can predict the response to immunotherapy in two melanoma cohorts but limites predictive efficacy was observed in other immunotherapy cohorts. D) The forest plot shows that the IGLoS score is associated with a trend toward a worse prognosis in more than half of the immunotherapy cohorts. E) The optimal predictor varied from cohort to cohort in the random forest model for predicting responses to ICI. The IGLoS score was among the top predictors in the IMvigor (muscle‐invasive urothelial carcinoma), GSE91061 (melanoma), and PRJNA482620 (glioblastoma) cohorts. The height of the bars represented the VIMP, with a large positive value indicating a more important predictor. The rank of the predictors indicates the minimal depth, with the minimal depth increasing from left to right. A lower minimal depth represents a more important predictor in the model. F) Representative IHC staining images of PDL1 from part of GBM patients in the Gusu in‐house dataset with higher IGLoS score and lower IGLoS score.

To assess the efficacy of the IGLoS score in predicting response to ICI, we calculated IGLoS scores for eight cohorts treated with ICI and grouped the patients into two IGLoS groups (**Figure** **5**A). As the IGLoS score increased, the GSVA scores for the IFNG signature, MHC I signature, and neoantigen signature increased, while the GSVA scores for oncogenic signaling pathways including the PI3K pathway, PTEN pathway, and Wnt pathway, decreased (Figure 5B). In addition, the ImmuneCellAI algorithm also returned predictions of the ICI response in four RNA‐seq cohorts of glioma, and the subclass mapping algorithm suggested that the high‐IGLoS group was likely to be made up of the patients predicted by the ImmuneCellAI algorithm to respond to ICI (Figure S6A, Supporting Information). The IGLoS score could predict response to immunotherapy in two melanoma cohorts, with more responders in the high‐IGLoS group in the PRJEB23709 and more responders in the low‐IGLoS group in the GSE78220; however, there was limited predictive efficacy in other immunotherapy cohorts (Figure 5C). The IGLoS score could also predict survival in patients receiving ICI and was a protective factor in the melanoma cohort PRJNA482620, with a trend toward worse prognoses in most of the other cohorts (Figure 5D). In the random forest model for predicting responses to ICI, the optimal predictor varied from cohort to cohort (Figure 5E). The IGLoS scores of the IMvigor (muscle‐invasive urothelial carcinoma), GSE91061 (melanoma) and PRJNA482620 (glioblastoma) cohorts were among the top three predictors of efficacy across the nine predictors, and we found that the enrichment score of CAFs was a very important predictor, recognized as the optimal predictor in PRJEB23709 (melanoma), GSE78220 (melanoma), and Braun (RCC).

Different markers are tissue‐agnostic measures of distinct aspects of tumor immunobiology, and they independently predict response to immunotherapy in multiple tumors^[^ ^28^ ^]^; thus, combinatorial strategies that capture attributes of the host and tumor‐immune ecosystem could have better predictive power.^[^ ^29^, ^30^ ^]^ We found that the predictive power of the combinatorial predictors was better than that of the individual predictors in eight immunotherapy cohorts and demonstrated a trend toward more accurate predictions with more predictors in the combination (Figure S6B, Supporting Information). Notably, the top five AUC‐ranked predictors in almost every cohort were combinatorial predictors that included the IGLoS score.

To validate the clinical utility of the IGLoS scoring system, we retrospectively analyzed tumor tissue from GBM patients in the Gusu in‐house dataset and categorized patients into high and low IGLoS groups based on their scores. Immunohistochemistry was performed to evaluate PD‐L1 expression in a subset of patients. Our results showed that patients with high IGLoS scores had significantly higher PD‐L1 expression levels than those with low scores, which was consistent with our findings and further supported the applicability of the IGLoS score in clinical settings (Figure 5F).

### 2.7 Exploration of Pharmacogenomics Helps Discover Promising Drug Candidates for Immunotherapy‐Resistant Patients with Glioma

**Figure 6:**
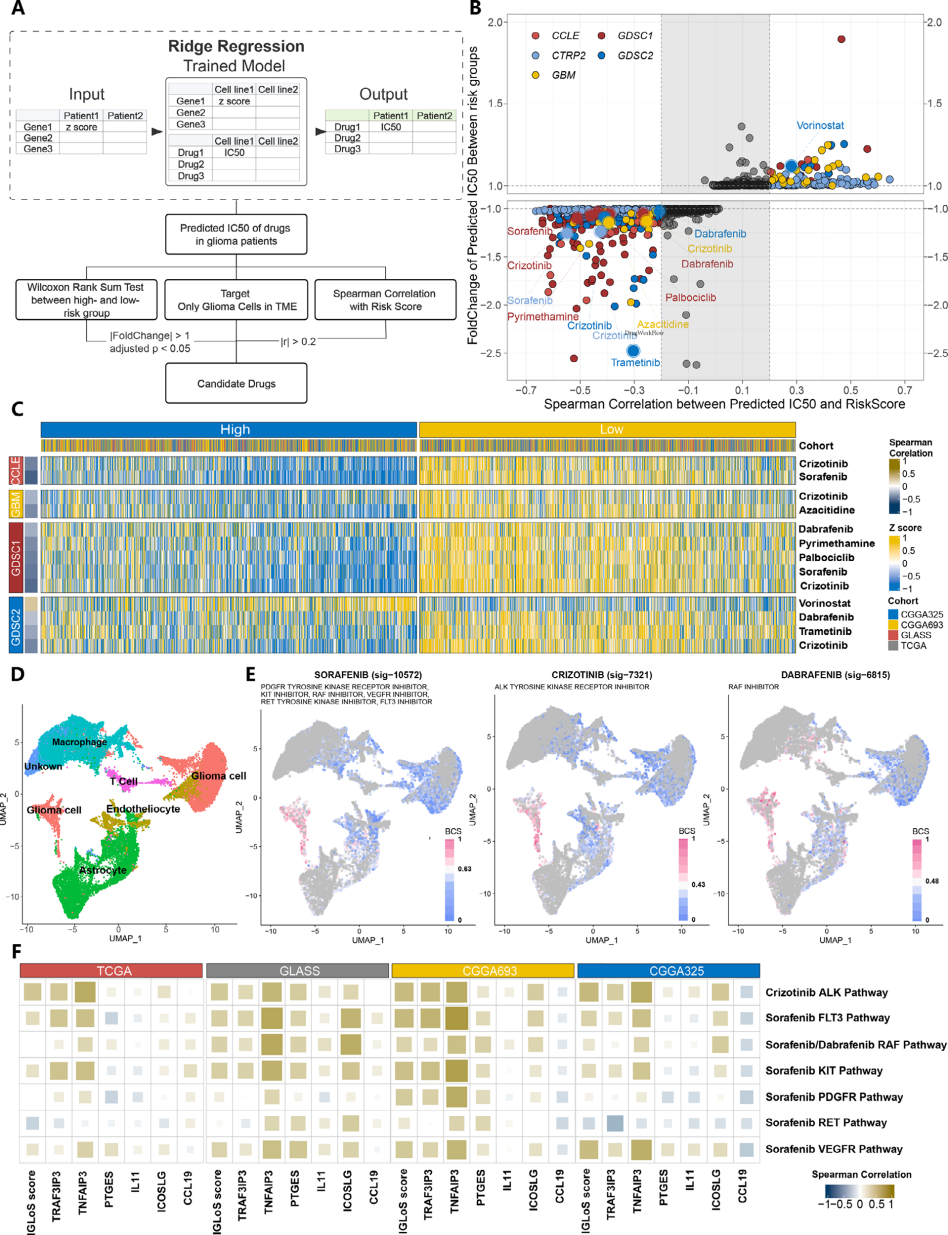
Predictive efficiency of the IGLoS score for drug therapy sensitivity in patients with glioma and screening of potential drugs for immunotherapy‐resistant patients. A) Summary of the pipeline for screening drug candidates in glioma. B) The dot plot showed 715 drugs with significantly different predicted IC_50_ and the absolute value of the correlation exceeding 0.2. Different colors represented different drug sensitivity datasets as training sets. C) The heatmap showed the predicted IC_50_ of eight drug candidates in four drug sensitivity datasets as training sets. D) Six clusters were identified through the UMAP reduction per the definition of marker genes. E) The UMAP plot identified the differences between glioma cells and other cells based on transcriptional changes induced by drugs from databases built in the “Beyondcell” package. A higher Beyondcell score indicates that the cell is more susceptible to the drug. F) Correlation between the IGLoS signature and the IGLoS score and targeted pathway activity of three drug candidates.

To assess the predictive power of the IGLoS score for drug therapy sensitivity in patients with glioma and to screen potential drugs for immunotherapy‐resistant patients, we calculated the predicted IC_50_ of 1177 small molecule drugs for 1817 patients with glioma, and then further screened the drug candidates. The screening process is summarized in **Figure** **6**A. Analyses of the difference in predicted IC_50_ values between the high‐IGLoS and low‐IGLoS groups showed that 926 of the 1177 drugs exhibited significantly different sensitivities between the two groups, with the absolute value of the correlation between 715 drugs and IGLoS scores exceeding 0.2 (Figure 6B). Of the 715 drugs screened, 513 had a predicted IC_50_ that was lower in the high‐IGLoS group, while 201 had a predicted IC_50_ that was lower in the low‐IGLoS group, suggesting that the high‐IGLoS group was more likely to benefit from chemotherapeutic agents and molecular targeted agents (Table S11, Supporting Information). Next, based on the single‐cell sequencing data of gliomas, we performed a further screening for 62 FDA‐approved drugs using the Beyondcell algorithm to identify eight drugs that specifically target glioma cells in the TME, including sorafenib, crizotinib, pyrimethamine, palbociclib, dabrafenib, vorinostat, trametinib, and azacitidine, and only vorinostat demonstrated better efficacy in the low‐IGLoS group of patients (Figure 6C). Among the eight drug candidates, sorafenib, crizotinib, and palbociclib were identified as drug candidates by IGLoS scores in at least two pharmacogenomic databases (Figure 6D,E, Figure S7, Supporting Information). In addition, based on the CMAP‐annotated mechanism of drug action, we extracted signatures of relevant pathways from MSigDB and calculated the corresponding GSVA scores in four glioma RNA‐seq cohorts, which were then correlated with the IGLoS signature (Figure 6F). We found that the IGLoS signature and the IGLoS score were roughly positively correlated with the activity of the three drug candidates' pathways of action, indicating that the higher the IGLoS score, the more sensitive the patient is to the drug.

Among the IGLoS signature, TRAF3IP3 showed the strongest positive correlation with these pathways, suggesting that it might be involved in the regulation of these pathways, Therefore, we hypothesized that TRAF3IP3 might bind directly to these three drugs, and we subsequently performed molecular docking to verify this hypothesis. The molecular docking simulations revealed comparable binding affinities of sorafenib, crizotinib, and palbociclib toward TRAF3IP3 (UniProt ID: Q9Y228), with calculated ΔG values ranging from −6.9 to −6.5 kcal mol^−1^. Among these compounds, sorafenib exhibited moderate binding affinity (ΔG = −6.5 kcal mol^−1^), while palbociclib demonstrated the strongest interaction (ΔG = −6.9 kcal mol^−1^) (Figure S8, Supporting Information).

### 2.8 The siRNA Screen Reveals that TRAF3IP3 Knockdown Downregulates PDL1 Expression and Activates Immune‐Related Pathways

**Figure 7:**
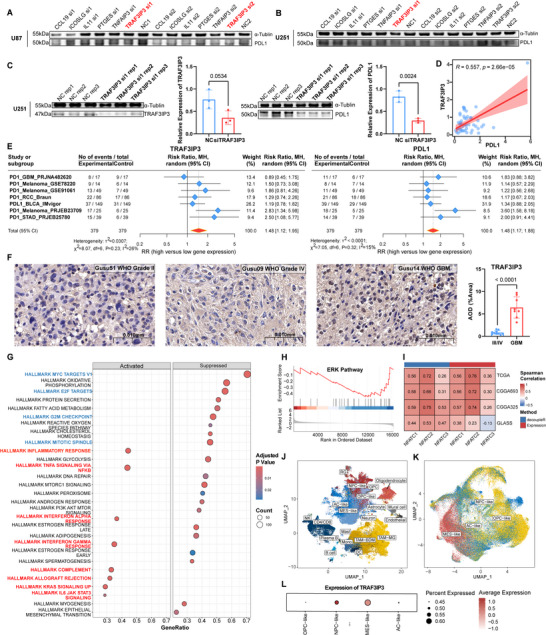
siRNA screen identifies TRAF3IP3 as downregulating PDL1 expression and activating anti‐tumor immune‐related pathways. A) Western blotting was used to detect the protein expression of PDL1 after the parallel knockdown of six genes in U87. B) Western blotting was used to detect the protein expression of PDL1 after the parallel knockdown of six genes in U251. C) Western blotting was used to detect the protein expression of TRAF3IP3 (left upper panel) and PDL1 (right upper panel) after TRAF3IP3 knockdown. The lower panel shows quantification of strips by ImageJ software (Statistic test: two‐sided unpaired t‐test. Data are presented as the mean ± 95% CI). D) Correlation analysis between the expression of TRAF3IP3 and PDL1 in the in‐house cohort. E) Forest plot showing the result of a meta‐analysis of response to immunotherapy for pan‐cancer cohorts for TRAF3IP3 (left panel) and PDL1 (right panel). F) Representative IHC staining images of TRAF3IP3 for glioma patients of WHO grade II (left panel), IV (middle panel) and GBM (right panel). G) Pathway enrichment analyses based on Hallmark gene sets. The bolded blue text represents proliferation and cell cycle‐related pathways, whereas the bolded red text represents immune‐related pathways. H) GSEA analysis of the ERK pathway based on differentially expressed genes from RNA‐seq after TRAF3IP3 knockdown. I) Correlation analysis between the expression of TRAF3IP3 and NFAT transcription factor family (NFATC1, NFATC2, NFATC3) quantified by expression and decoupleR algorithm. J) The UMAP reduction revealed 20 distinct clusters of in‐house cohort. K) The UMAP reduction revealed four distinct subtypes of malignant cells: AC‐like, MES‐like, NPC‐like and OPC‐like. L) Bubble plot showing mean expression of TRAF3IP3 in distinct subtypes.

In our analysis of the correlation between IGLoS and the expression of 74 key immune modulators (Figure 2F), we found a consistent and highly positive correlation between the IGLoS signature and the PD1 (PDCD1) /PDL1 (CD274) axis, which has been widely proven to play a vital role in the maintenance of the anti‐tumor immune.^[^ ^31^ ^]^ Therefore, we designed two siRNA sequences for each of the six genes in the IGLoS signature, to further screen targets that could regulate the PDL1 expression of glioma. We parallelly knocked down the six genes in the U87 and U251 cell lines and found that PDL1 was significantly downregulated in U87 (Figure 7A) and U251 (Figure 7B) when TRAF3IP3 was knocked down. To demonstrate that TRAF3IP3 was indeed knocked down when transfecting specific siRNAs into the glioma cells, we performed three biological replicates in a new round of transfection in U251, finding that TRAF3IP3 was knocked down and PDL1 was downregulated (Figure 7C), which was validated in our in‐house cohort (Figure 7D). To explore the relationship between the expression of TRAF3IP3 and response to immunotherapy, we performed meta‐analyses in seven independent cohorts receiving anti‐PD1/PDL1 therapy for TRAF3IP3 and PDL1, and the results showed that TRAF3IP3 could act as an indicator for immunotherapy, as well as the widely acknowledged PDL1 (Figure 7E). In addition, immunohistochemistry of the glioma tissues from our in‐house cohort showed an upregulation of TRAF3IP3 as the tumor grade increased (Figure 7F, Figure S9), which was in accordance with observations at the transcriptomic level (Figure S3C). To explore the mechanism by which TRAF3IP3 knockdown downregulated PDL1, a 3v3 RNA‐seq was performed. PCA and inter‐sample correlation analyses showed significant differences between TRAF3IP3‐knockdown cells and the control group (Figure S10A‐B), and differential expression analyses yielded 370 up‐regulated and 220 down‐regulated genes in the TRAF3IP3‐knockdown cells, respectively (Figure S10C). Then, pathway enrichment analysis was performed based on hallmark gene sets and KEGG gene sets. In Hallmark, pathways associated with anti‐tumor immunity (inflammatory response, TNFA signaling via NFKB, IFNA response, IFNG response, and so on) were significantly activated while pathways associated with proliferation and the cell cycle (MYC targets, E2F targets, G2M checkpoint, mitotic spindle, and so on) were significantly inhibited (Figure 7G), and the same trend was observed in KEGG (Figure S10D‐E).

### 2.9 TRAF3IP3 may Activate the ERK Pathway to Initiate Transcriptional Activation of PDL1 by NFATC2

During the development of T lymphocytes, TRAF3IP3 was proven to mediate TCR‐stimulated activation of the mitogen‐activated protein kinase (MAPK) extracellular signal‐regulated kinase (ERK) and its upstream kinase mitogen/extracellular signal‐regulated kinase (MEK).^[^ ^32^ ^]^ Lin et al. found that overexpressed TRAF3IP3 could promote glioma progression through the ERK signaling pathway,^[^ ^33^ ^]^ which was supported by GSEA analysis of the ERK pathway in this study (**Figure** 7H). According to the annotation of PD‐L1 expression and PD1 checkpoint pathway in cancer in the KEGG database (Figure S11, Supporting Information), the nuclear factor of activated T‐cells (NFAT) transcription factor family (NFATC1, NFATC2, and NFATC3) could mediate the transcriptional activation of PDL1 in the tumor cells, which was activated by ERK pathway. The correlation analysis between the expression of TRAF3IP3 and the activity of the NFAT transcription factor family showed that the transcriptomic activation of PDL1 was most likely initiated by NFATC2 (Figure 7I). Considering the heterogeneity of tumor cells in the glioma TIME, we specifically extracted four subtypes of tumor cells: OPC‐like, MES‐like, NPC‐like, and AC‐like cells from our in‐house dataset and found that TRAF3IP3 exhibited selective enrichment in NPC‐like and MES‐like tumor cells (Figure 7J‐L). PDL1 expressed on the surface of tumor cells has been widely demonstrated to inhibit T cell functionality through the PD1/PDL1 axis, which is a canonical pathway for T cell exhaustion and results in decreased cytotoxic T‐lymphocyte‐mediated lysis to allow for glioma cell immune evasion.^[^ ^34^ ^]^ Therefore, we hypothesize that TRAF3IP3 in glioma cells upregulates PDL1 expression through transcriptional activation of PDL1 by NFATC2 via the ERK pathway, leading to the depletion of CD8+ T cells in the TME, which results in the overall suppression of the TME and, thus, resistance to immunotherapy (Figure S12, Supporting Information).

### 2.10 Unveiling the Dual Functionality of TRAF3IP3 in Mediating T‐Cell Exhaustion through Bulk and Single‐Cell Resolution Analyses

To preliminarily verify our hypothesis, as well as to further elucidate the role of TRAF3IP3 in immune escape, we conducted further analyses of the association of TRAF3IP3 with T‐cell exhaustion at bulk RNA sequencing resolution and single‐cell resolution. At the bulk level, we identified a series of regulators and pathways that are integral to the process of T cell exhaustion. By quantifying pathway scores across four cohorts, we found that most of these indicators representing the degree of T cell exhaustion showed significant differences with increasing TRAF3IP3 expression, highlighting the potential role of TRAF3IP3 in mediating T‐cell exhaustion (Figure S12A, Supporting Information). At the single‐cell level, we annotated three previously published datasets and our in‐house dataset using reference mapping based on GBmap, which provides a comprehensive harmonization of the single‐cell landscape, intercellular crosstalk, and tumor architecture within glioblastoma. Unexpectedly, TRAF3IP3 was found to be specifically expressed in lymphocytes, rather than the tumor cells that were the focus of our study (Figure S11B), Supporting Information. This observation prompted us to re‐evaluate our screening process, including the prognostic meta‐analysis and the statistical significance of TRAF3IP3 as an important feature identified by machine learning. This observation highlights the potential of TRAF3IP3 not only for its role in tumor cells as demonstrated in this study. The strong statistical significance and findings from the prognostic meta‐analysis and machine learning position TRAF3IP3 as a more promising target, which opens up an exciting new direction for further investigation. The coexistence of potential crucial roles for regulators in both tumor cells and T cells has been validated by recent research for TNFAIP3, which has been identified as a key player in enhancing cancer cell susceptibility to immune attack and promoting T cell activation.^[^ ^35^ ^]^ Hence, we are justified in postulating that TRAF3IP3 similarly manifests a dual functionality within these cellular contexts.

## 3 Discussion

To date, as limited patient stratification methods were available to rationally select patients who might benefit most from treatment, the efficacy reported so far in gliomas is poor.^[^ ^7^, ^36^ ^]^ We hypothesized that integrative analyses of large‐scale data would be helpful for comprehensively exploring glioma–immune cell interactions and developing reliable tools for precision medicine. In this study, we separately integrated transcriptomic data from four RNA‐seq glioma cohorts and four microarray glioma cohorts to identify a robust IGLoS signature. Then we developed a pipeline consisting of 18 finely tuned ML models, and with the expression profiles of the IGLoS signature and survival data, the tuned Gradient Boosting model showed the highest c‐index and was selected to calculate the IGLoS score. The IGLoS score showed a robust predictive power for survival in glioma as well as the TCGA pan‐cancer cohorts. Furthermore, the IGLoS score could depict the cross‐talk between immune cells and glioma cells, predict the response to ICI in pan‐cancer immunotherapy cohorts including glioma, and help to discover promising drug candidates for immunotherapy‐resistant patients. Through further siRNA screen, TRAF3IP3 of the IGLoS signature was proven to be a promising target to improve the resistance to immunotherapy in glioma.

The IGLoS signature consists of six immune infiltration‐related genes associated with the survival of diffuse patients with glioma, which are CCL19, ICOSLG, IL11, PTGES, TNFAIP3, and TRAF3IP3. CCL19 (CC‐chemokine ligand 19) and CCL21 (CC‐chemokine ligand 21) are the sole ligands for the CCR7 (CC‐chemokine receptor 7), which is expressed by various subsets of immune cells.^[^ ^37^ ^]^ IFN‐α‐induced dendritic cells of patients with malignant gliomas, despite preserved expression of CCR7, are characterized by lower migration activity in response to CCL19 and CCL21 stimulation.^[^ ^38^ ^]^ Inducible co‐stimulator‐ligand (ICOSLG), a member of the B7 family of costimulatory molecules related to CD80/CD86, regulates CD4 as well as CD8 T‐cell responses via interactions with its receptor, ICOS, on activated T cells.^[^ ^39^ ^]^ ICOSLG has been proven to be highly expressed in malignant gliomas by in vivo and in vitro experiments and mediates Treg expansion^[^ ^40^ ^]^ and IL‐10 production to promote the progression of glioblastoma.^[^ ^41^ ^]^ Interleukin‐11 (IL11), a member of the IL‐6 cytokine superfamily, is a pleiotropic cytokine that binds to its specific receptor (IL11Rα) and the transmembrane co‐receptor gp130.^[^ ^42^ ^]^ Stuart et al. found that the IL11/IL11Rα signaling axis plays a critical role in glioblastoma survival, proliferation, and invasion when cells are starved of glucose,^[^ ^43^ ^]^ and Li et al. proven that glioblastoma‐associated microglia/TAM secrete high levels of the proinflammatory cytokine IL11, which in turn, triggers the STAT3‐MYC signaling axis in glioblastoma cells to induce stem cell states that confer temozolomide resistance.^[^ ^44^ ^]^ Microsomal prostaglandin E2 synthase‐1 (mPGES‐1), also known as PTGES, is the rate‐limiting enzyme responsible for the synthesis of prostaglandin E2 (PGE2) downstream of COX‐2.^[^ ^45^ ^]^ Lalier et al. found an increased expression of mPGES‐1 in glioblastoma, and the sensitivity of primary cultures of GBM to apoptosis was augmented by the overexpression of mPGES‐1.^[^ ^46^ ^]^ Inhibition of COX‐2, mPGES‐1, and CYP4A by isoliquiritigenin can block the angiogenic Akt signaling in glioma through ceRNA effect of miR‐194‐5p and lncRNA NEAT1 (Inhibition of COX‐2, mPGES‐1, and CYP4A by isoliquiritigenin blocks the angiogenic Akt signaling in glioma through the ceRNA effect of miR‐194‐5p and lncRNA NEAT1).^[^ ^47^ ^]^ Tumor necrosis factor alpha‐induced protein 3 (TNFAIP3, also known as A20), which acts in a negative feedback loop to block NF‐κB activity and is significantly associated with resistance to TMZ, is frequently downregulated in glioblastoma cells.^[^ ^48^ ^]^ Tumor necrosis factor receptor‐related factor 3 (TRAF3) interacting protein 3 (TRAF3IP3) can specifically interact with TRAF3 and then mediate the activation of the JNK signaling pathway.^[^ ^49^ ^]^ TRAF3IP3 was identified as a key gene by Lin's bioinformatic analysis and was proven to promote glioma progression through the ERK signaling pathway by experiments.^[^ ^33^ ^]^ Although researchers have conducted in‐depth mechanistic investigations of ICOSLG, IL11, PTGES, and TNFAIP3 in gliomas, the roles of CCL19 and TRAF3IP3 in glioma need to be further characterized.

Glioma is widely recognized as an immune‐cold tumor.^[^ ^50^ ^]^ However, based on our prognostic analysis and findings on immune infiltration, we indeed observed that glioblastoma patients with relatively higher IGLoS scores exhibited poorer outcomes, and this group also showed a higher quantitative level of immune cells. This result could suggest that “immune‐cold tumors have superior prognostic outcomes,” which contrasts with clinical observations that report poor prognoses for immune‐cold tumors. One possible reason might be that a significant portion of the immune cells in the glioma TME are exhausted, or “hijacked,” not only the T‐cells as shown in Figure S5C (Supporting Information) but also macrophages, and neutrophils, among others. In addition, patients in the high‐IGLoS group showed weaker intrinsic resistance mechanisms (Figure 5B), suggesting that the application of immunotherapy to these patients could provide greater benefit. We must acknowledge that analysis of this part has remained at a somewhat superficial level, merely stating that “immune‐related pathways are more active in patients with higher IGLoS scores”, without delving into specific cell types. We believe that the resolution of bulk RNA sequencing is insufficient to support a deeper exploration of the interactions among immune cells in the microenvironment. However, further investigation at the single‐cell resolution here might divert attention from our central focus—characterizing the association between IGLoS scores derived from bulk RNA sequence and immune activity. Therefore, we tentatively assert that “increased correlation simply indicates a higher probability that an actual association may exist”, and refrain from making further specific inferences.

Recently, the amount of transcriptomic data with response information of cancer immunotherapy has grown rapidly, which could provide the basis for large‐scale discovery and ranking of such potential biomarker candidates.^[^ ^51^ ^]^ Some markers have been used in clinical practice, such as PD‐L1, MSI‐H, TMB, and GEP^[^ ^28^ ^]^; however, they seem to have difficulty in predicting glioma response to immunotherapy.^[^ ^19^ ^]^ To date, two known studies have developed novel markers based on large‐scale glioma transcriptomic data and validated them in pan‐cancer immunotherapy cohorts^[^ ^24^, ^52^ ^]^; however, our results were not the same as the findings of either study. In our research, the higher the IGLoS score, the worse the prognosis for patients with glioma and the worse the prognosis for the pan‐cancer immunotherapy cohort, but the more likely they were to benefit from immunotherapy. Our results might be more plausible because factors contributing to ICI resistance^[^ ^29^ ^]^ were all attenuated in patients with glioma with increasing IGLoS score (Figure 5B). Furthermore, our study first constructed 31 combinatorial strategies of transcriptomes, and the results showed that the combinatorial strategy was indeed superior to a single predictor, while the IGLoS score was an important component in the combinatorial strategies. However, there are very few publicly available glioma immunotherapy cohorts currently, which makes external validation of a large‐scale ML pipeline based on glioma immunotherapy responses more difficult. We plan to request sequencing data and response information from centers with published glioma immunotherapy cohorts to develop an ML pipeline for large‐scale classification tasks, which could lead to the development of more accurate markers of immunotherapy response in glioma.

In our study, large‐scale drug sensitivity datasets were used to build the ridge regression model, which was then applied to transcriptomic datasets of glioma to yield drug sensitivity predictions for patients with glioma. scRNA‐seq has become an established piece of technology to dissect tumor heterogeneity at the transcriptional level, making it possible to screen for drugs that target glioma cells specifically in the TME.^[^ ^53^ ^]^ Based on the drug screening pipeline we built, sorafenib, crizotinib, and palbociclib were selected as promising drug candidates for immunotherapy‐resistant patients with glioma. Sorafenib is an inhibitor of wild‐type and mutant Raf‐kinase, and it inhibits other tyrosine kinases related to tumor progression, including VEGFR‐2, flt‐3, c‐KIT, and MAPK.^[^ ^54^ ^]^ It was initially used to treat malignant glioma in 2011 because a large number of target kinases are activated in malignant gliomas.^[^ ^55^ ^]^ However, significant side effects and moderate outcomes resulted in the following clinical trials do not support sorafenib's further clinical development in malignant gliomas.^[^ ^56^, ^57^ ^]^ Crizotinib is a potent inhibitor of ALK and c‐Met, and it was used in combination with dasatinib in a phase I clinical trial for the treatment of pediatric HGG and DIPG^[^ ^58^ ^]^; however, this combination was poorly tolerated and its activity was minimal, thus preventing further clinical trials of crizotinib in gliomas. Palbociclib is an oral inhibitor of cyclin‐dependent kinases (CDKs) 4 and 6, and it is used in preclinical models of glioma. The CDK4‐cyclin D1 axis is critical for glioma genesis, tumor de t velopment, and the growth of stromal‐derived cells in the surrounding microenvironment, which sustains the spread of glioma cells.^[^ ^59^ ^]^ Despite its good tolerance, palbociclib monotherapy did not show favorable efficacy against recurrent anaplastic oligodendroglioma and recurrent glioblastoma.^[^ ^60^, ^61^ ^]^ Although none of the three drugs have entered further clinical trials, all have moved from preclinical to clinical trials, suggesting that our screening pipeline is reasonable. In addition, we demonstrated by molecular docking that all three drugs could directly bind to the protein of TRAF3IP3 in the IGLoS signature, with the most significant binding by Palbociclib. Although further experimental validation is needed to elucidate whether sorafenib or other drugs modulate TRAF3IP3‐mediated immune resistance via ERK/NFATC2/PD‐L1 signaling, the result once again confirms the reliability of our drug screening process based on the IGLoS score and the potential of TRAF3IP3 in glioma‐targeted therapies. Thus, it is reasonable to assume that some of the drugs screened but not yet approved by the FDA may hold promise for glioma patients.

## 4 Conclusion

In conclusion, we developed a novel pipeline of 18 finely tuned ML models based on nested resampling and cross‐validation, and by integrative analysis of multi‐omics data of diffuse glioma, a robust IGLoS score was constructed to accurately stratify diffuse patients with glioma. A higher IGLoS score may indicate a worse prognosis and an “immune‐hot” phenotype that can benefit from immunotherapy, chemotherapeutics, and molecular targeted therapy, which was validated at the pan‐cancer level. TRAF3IP3 of the IGLoS signature is a promising target to improve the resistance to immunotherapy in glioma.

## 5 Experimental Section

### Bulk Transcriptomic Data of Glioma Acquisition and Preprocessing

In total, 2968 diffuse glioma patients with complete survival information from eight independent public datasets were accessed. The RNA‐seq raw read count was converted to transcripts per kilobase million (TPM) and further log‐2 transformed because they were more similar to gene expression from microarrays and enhance inter‐sample comparability.^[^ ^62^ ^]^ Data from the GEO database was retrieved from the Affymetrix GPL570 (Rembrandt cohort and Kamoun cohort) and Affymetrix GPL8542 (Gravendeel cohort) platforms. The raw data from Affymetrix were processed via the robust multiarray averaging (RMA) algorithm implemented in the “Affy” package. When multiple probes were mapped to a single gene symbol, the probe with the highest expression was annotated as gene expression. For the merging of different datasets to a meta cohort, the “ComBat” function in the “sva” package was used to adjust for batch effects from non‐biological technical biases of each dataset using an empirical Bayes framework.^[^ ^63^ ^]^ In modules using machine learning algorithms, each gene expression was transformed into the z‐score across patients. To further prove the rationality of data merging and reduce heterogeneity between glioma cohorts for the following meta‐analysis, the tSNE algorithm was performed to visualize the consistency across glioma cohorts before and after merging.

### Multi‐Omics Landscape at the Pan‐Cancer Level

At the genomic level, the single‐nucleotide variation (SNV, including seven types of variation: Missense Mutation, Nonsense Mutation, Frame Shift Ins, Splice Site, Frame Shift Del, In Frame Del and In Frame Ins) data of 10 234 samples from 33 cancer types were collected from the Gene Set Cancer Analysis^[^ ^64^ ^]^ portal (GSCA, http://bioinfo.life.hust.edu.cn/GSCA/#/, 33 types of cancer). At the epigenetic level, the Illumina HumanMethylation 450k level 3 data was analysed from the GSCA portal. The methylation level of each probe was measured as the beta value, which was calculated as the ratio of the methylated signal to the sum of the methylated and unmethylated signals, and the beta value ranges from 0 (unmethylated) to 1 (completely methylated). For each gene, the methylation site most negatively correlated with gene expression was selected to fit the univariate Cox regression in patients with overall survival (OS) or progression‐free survival (PFS). At the transcriptomic level, the univariate Cox regression and differential expression analyses were performed.

At the immunogenomic level, the pre‐calculated T cell dysfunction score using the interaction test model and the correlation between the level of cytotoxic T lymphocytes and gene expression were accessed from the Tumor Immune Dysfunction and Exclusion portal^[^ ^65^ ^]^ (TIDE, http://tide.dfci.harvard.edu/, 14 types of cancer). In addition, the “Gene set prioritization module” of the TIDE portal was used to evaluate each gene for its expression associations with ICI response outcome, T cell dysfunction levels, T cell exclusion levels, and phenotypes in genetic screens in diverse cohorts.^[^ ^66^ ^]^

### Meta‐Analysis for Prognostic Genes in Glioma

The meta‐analysis was conducted using the “meta” package.^[^ ^67^ ^]^ To reduce heterogeneity across cohorts, each log‐2 transformed gene expression was transformed into the z‐score across patients. The univariate Cox regression analysis was used to obtain the Benjamini‐adjusted hazard ratio (HR) and 95% confidence interval (CI) of each gene in individual cohorts, based on which the meta‐analysis for each gene was performed across four RNA‐seq cohorts and four microarray cohorts, respectively. The random‐effect model was used to calculate the summary statistic HR and 95% CI and *p*‐value < 0.05 was considered statistically significant.

### Characterization of the Landscape of TME

To characterize a comprehensive landscape of TME, a systematic exploration of published literature was performed, at the end of which 17 immune cell signatures and 15 immune function signatures were included. The majority of signatures were extracted from searches of He^[^ ^68^ ^]^ and White,^[^ ^10^ ^]^ and signatures depicting the activity of IFNG,^[^ ^69^ ^]^ cytotoxic activity (CYT),^[^ ^70^ ^]^ T‐cell exhaustion,^[^ ^71^ ^]^ and T cell‐inflamed gene expression profile (GEP)^[^ ^28^ ^]^ were extracted from other high‐quality searches. Finally, after deleting the overlaps between selected signatures, 606 immune‐infiltration‐related genes were obtained and their details are shown in Table S2 (Supporting Information).

### Integrative Pipeline of 18 Machine Learning Algorithms

To select features, the Least Absolute Shrinkage and Selection Operator (LASSO) regression was performed by the “glmnet” package to screen the most important features from genes that were identified as both prognostic and immune infiltration‐related genes. To enhance the interpretability of the model, relative influence was computed by the reduction of squared error attributable to each variable normalized to sum to 100, and marginal effect change was selected as a particular feature while averaging over or integrating out the influence of all other features. To compare the generalized performance of the 18 included models,a nested resampling was incorporated with a 10‐fold inner loop and a 5‐fold outer loop for hyperparameter optimization (HPO) and model benchmarking using selected features by the “mlr3” and “mlr3proba” packages.^[^ ^72^, ^73^ ^]^ The hyperparameter was tuned in the inner loop with the Bayesian optimization approach optimized for low‐dimensional data. In brief, Kriging regression was used as a surrogate mode with a 20‐iteration random search evaluated by expected improvement. The final benchmark was generated by integrating the C‐index of the nested performance over the outer iterations using the selected best hyperparameter set in hyperparameter tuning. The glioma Meta‐cohort was then partitioned into a training set and a test set in a 2:3 ratio. Next, the best model was trained with the training set of the Meta‐cohort with hyperparameters set selected in the HPO process and then validated the trained model with the test set of the Meta‐cohort. Based on the model benchmark, the fine‐tuned model with the highest C‐index was fitted on the glioma Meta‐cohort, immunotherapy cohorts, and TCGA pan‐cancer cohorts to calculate the IGLoS score and stratify patients into high‐IGLoS and low‐IGLoS groups.

### The Multi‐Omics Landscape of the IGLoS Score in Glioma

Multi‐omics data of TCGA‐LGG and TCGA‐GBM were accessed using the “TCGAbiolinks” package.^[^ ^74^ ^]^ At the genomic level, the “maftools” package^[^ ^75^ ^]^ was used to analyze the SNV data. At the epigenetic level, the “ChAMP” package^[^ ^76^ ^]^ was used to identify differentially methylated probes between different groups with adjusted *p* values of < 0.05 and then performed the univariate Cox regression to access the predictive ability of differentially methylated CpG probes to determine the prognosis. The “IlluminaHumanMethylation −450kanno.ilmn12.hg19” package was incorporated to annotate the methylation probe, including the genomic region [regions from 1500 bp upstream to the transcription start site (TSS1500), regions from 200 bp upstream to the transcription start site (TSS200), 50 UTR, 1stExon, Body and 30UTR] and CpG island (CGI)‐associated regions [Island, Shore (up or down 2 kb from CGI), Shelf (up or down 2–4 kb from CGI), and Open sea (> 4 kb from CGI)]. At the transcriptomic level, the “DESeq2” package^[^ ^77^ ^]^ was used to identify differentially expressed genes between different groups, based on which Gene Set Enrichment Analysis (GSEA) and Kyoto Encyclopedia of Genes and Genomes (KEGG) enrichment analysis was performed using the “clusterProfiler” package.^[^ ^78^ ^]^

### Comprehensive Analysis of Immunogenomic Molecular Characterization

IOBR was an R package used to perform comprehensive analyses of the tumor microenvironment and signatures for immuno‐oncology,^[^ ^79^ ^]^ of which MCP‐counter (8 cell types), xCell (64 cell types), quanTIseq (10 cell types), CIBERSORT (22 cell types), and EPIC (6 cell types) were performed to evaluate the different levels of immune cell infiltration. The anti‐cancer immune response can be conceptualized as a series of stepwise events referred to as the Cancer‐Immunity Cycle, and the TIP (Tracking Tumor Immunophenotype, http://biocc.hrbmu.edu.cn/TIP/) server was used to analyze the activity of seven‐step Cancer‐Immunity Cycle based on four RNA‐seq cohorts.^[^ ^80^ ^]^ The ImmuneCellAI portal (Immune Cell Abundance Identifier, http://bioinfo.life.hust.edu.cn/ImmuCellAI#!/) was used to estimate the abundance of 18 T‐cell subtypes and six other immune cells.^[^ ^81^ ^]^Signatures of key pathways were collected in cancer immunology from previous publications, including angiogenesis, CYT, GEP, T‐cell exhaustion, and tertiary lymphoid structure (TLS) (Table S2, Supporting Information).

### The prediction of ICI Response at the Pan‐Cancer LEVEL

In the glioma Meta cohort, the Gene Set Variation Analysis (GSVA) algorithm was used to depict the activity of tumor cells' intrinsic pathways causing resistance to ICI via the “GSVA” package,^[^ ^82^ ^]^ and signatures related to these pathways were collected from the Molecular Signatures Database^[^ ^83^ ^]^ (MSigDB, https://www.gsea‐msigdb.org/gsea/msigdb) (Table S3, Supporting Information). The ImmuneCellAI portal was used to predict the ICI response of patients with glioma and the subclass mapping algorithm was used to evaluate the consistency between the predicted response and IGLoS groups.^[^ ^84^ ^]^

After removing batch effects, log‐2, and z‐score transformation, the IGLoS score was calculated in each immunotherapy cohort. The univariate Cox regression was used to evaluate the prognostic potential of the IGLoS score in immunotherapy cohorts. The “RandomForestSRC” package (https://www.randomforestsrc.org/) was used to construct random forests for classification tasks, in which the label was the response to ICI, and the features were widely acknowledged predictors of the ICI response derived from the TIDE portal, including the TIDE score, IFNG (IFNG average expression of interferon‐gamma response signature), the MSI score (microsatellite instability score predicted through gene expression), CD274 (gene expression of CD274), CD8 (gene expression average of CD8A and CD8B), and the enrichment scores of immunosuppressive cell types consisting of MDSC (myeloid‐derived suppressor cell), CAF (cancer‐associated fibroblast), and TAM M2 (tumor‐associated macrophage M2 type). The “tune” function was used to find the optimal “mtry” and “nodesize” tuning parameters using out‐of‐sample error before fitting the model, and the number of trees was set to 1000. Permutation importance (referred to as Breiman‐Cutler importance) was used to quantify the Variable Importance (VIMP), and a large positive value indicates a more important predictor. The “var.select” function was used to select important features based on a tree‐based concept called minimal depth, and a smaller depth indicates a variable with predictive importance. Individual predictors in isolation were insufficient, and combinatorial strategies that capture features of the host and tumor‐immune ecosystem have better predictive power.^[^ ^83^ ^]^ A total of 31 combinatorial strategies based on five transcriptomic predictors (IGLoS score, MSI score, IFNG score, CD8, and CD274) were generated, and the area under the ROC curve (AUC) was calculated for comparison between individual predictors and combinatorial strategies.

### Integrative Pharmacogenomic Sight into Potential Drugs for Glioma

The “OncoPredict” package was used to predict in vivo drug responses in cancer patients.^[^ ^85^ ^]^ The “calcPhenotype” function can predict the half‐maximal inhibitory concentration (IC_50_) of drugs in patients with glioma by fitting a ridge model, in which the train sets were gene expression profiles of tissues and IC_50_ of the cancer cell lines to drugs from pharmacogenomic datasets, and the testing sets were four RNA‐seq cohorts. The final drug candidates needed to fulfill the following criteria: 1) FDA‐approved, identified by the FDA Drugs Database (https://www.drugfuture.com/fda/); 2) target only glioma cells in TME, identified by the “Beyondcell” package using glioma scRNA‐seq data^[^ ^53^ ^]^; 3) the significant difference of predicted IC_50_ between the low‐IGLoS and high‐IGLoS groups, with a threshold adjusted *p*‐value of < 0.05 per wilcoxon signed‐rank test; 4) a strong correlation between the predicted IC_50_ and the IGLoS score by the Spearman correlation analysis. The action pathways of drug candidates were annotated using the Connectivity Map (CMAP, https://www.broadinstitute.org/connectivity‐map‐cmap), characterized using the MSigDB database, and quantified using the GSVA algorithm.

### Construction of Gusu in‐House Cohort

Glioma samples were obtained from patients who underwent neurosurgical resection at the First Affiliated Hospital of Soochow University. All specimens underwent high‐throughput sequencing, with a subset of these samples further analyzed through single‐cell transcriptomic sequencing (Table S5, Supporting Information). The sequencing services were performed by OE Biotech Co., Ltd. (Shanghai, China). This study complied with the Declaration of Helsinki and was approved by the Medical Ethics Committee of the First Affiliated Hospital of Soochow University (Approval No. 247). Written informed consent was acquired from all participating patients prior to sample collection.

### Cell Culture and Transfection

The U87 and U251 cells were cultured in RPMI 1640 medium (cat. no. RG‐CE‐4, Ketu Biotech) containing 10% fetal bovine serum (FBS) (cat. no. 35 050 061, Gibco) at 37 °C and 5% CO_2_. Twelve siRNAs (Small interfering RNAs, two siRNA sequences for each of the six genes in the IGLoS signature) were purchased from Tsingke (Beijing, China). The U87 and U251 cells were seeded in six‐well plates, and siRNA transfection experiments were performed when the cell density reached 70–80% (cat. no. L3000001 Invitrogen). Transfection was performed according to the manufacturer's instructions. Protein was extracted 48 h after transfection for subsequent Western Blotting. The siRNA sequence is shown in Table S4 (Supporting Information).

### Western Blot

Cells were collected and fully lysed with the RIPA lysis buffer containing 1% phenylmethanesulfonyl fluoride (cat. no. P0013B, Beyotime). A protein loading buffer was then added to the protein sample and boiled in a 95 °C water bath for 5 min, after which the protein was subjected to SDS‐PAGE and transferred to a polyvinylidene fluoride (PVDF) membrane after electrophoresis. Then, the membranes were blocked with a blocking buffer (cat. no. P0023B, Beyotime) for 1 h and incubated with the following primary antibodies overnight at 4 °C: anti‐PDL1 (1:1000; cat. no. 66248‐1‐Ig; Proteintech) and anti‐TRAF3IP3 (1:1000; cat. no. 18110‐1‐AP; Proteintech). HRP‐conjugated alpha‐tublin (1:10 000; cat. no. HRP‐66031; Proteintech) was set as the loading control. The membranes were washed thrice for 5 min in a mixture of TBST, incubated with HRP‐conjugated anti‐mouse IgG secondary antibodies (1:10 000; cat. no. SA00001‐1; CST) and HRP‐conjugated anti‐rabbit IgG secondary antibodies (1:10 000; cat. no. SA00001‐2; CST) for 2 h, and washed again thrice for 5 min in TBST. Immunoblotting bands were observed using the ChemiDoc XRS+ System (Bio‐Rad) after incubation with the Pierce ECL Plus Western Blotting Substrate (cat. no. P2100; Ncmbio). The intensity of the western blotting bands was measured using ImageJ software.

### Immunohistochemistry

Paraffin‐embedded tumor tissues of glioma samples used for RNA sequencing from the Gusu in‐house dataset were further collected for IHC staining. The tissues were fixed in a 4% PFA fix solution and embedded in paraffin. The paraffin‐embedded tissues were sectioned and stained with hematoxylin (cat. no. G1005; Servicebio). Immunohistochemistry staining of TRAF3IP3 (1:1000; cat. no. 18110‐1‐AP; Proteintech) and PD‐L1 (1:500; 1:1000; cat. no. 66248‐1‐Ig; Proteintech) was performed per the manufacturer's protocols for the Immunohistochemical assay kit (cat. no. PK10006; Proteintech).

### RNA Sequencing and Downstream Analysis

Total RNA was extracted using the TRIzol reagent (Invitrogen) per the manufacturer's protocol. RNA purity and quantification were evaluated using the NanoDrop 2000 spectrophotometer (Thermo Scientific). RNA integrity was assessed using the Agilent 2100 Bioanalyzer (Agilent Technologies). Then, the libraries were constructed using VAHTS Universal V6 RNA‐seq Library Prep Kit per the manufacturer's instructions. The libraries were sequenced on an llumina Novaseq 6000 platform, and 150 bp paired‐end reads were generated. Raw reads of the fastq format were mapped to the reference genome using STAR.^[^ ^86^ ^]^

Principal components analyses (PCAs) were performed to evaluate the biological duplication of samples by the “ggbiplot” package. The differential expression analysis was performed using DESeq2. An FDR adjusted *p*‐value of < 0.05, a Q‐value < 0.05, and foldchange > 2 or foldchange < 0.5 were set as the thresholds for the significance of differentially expressed genes (DEGs). The Gene Set Enrichment Analysis (GSEA), KEGG, and HALLMARK enrichment analyses were performed using the “clusterProfiler” package.^[^ ^87^ ^]^ Transcription factor activity was inferred by the “decoupleR” package from prior knowledge.^[^ ^88^ ^]^

### Single‐Cell Transcriptome Sequencing Analysis

Single‐cell transcriptomes of glioma samples from previously published datasets were downloaded from the GBmap,^[^ ^89^ ^]^ as well as GSE154795 (https://www.ncbi.nlm.nih.gov/geo/query/acc.cgi?acc = GSE154795), GSE167960 (https://www.ncbi.nlm.nih.gov/geo/query/acc.cgi?acc = GSE167960), and GSE174554 (https://www.ncbi.nlm.nih.gov/geo/query/acc.cgi?acc = GSE174554), and our in‐house cohort consists of 16 patients diagnosed as glioma (Table S5, Supporting Information). GBmap offers an extensive normalization of the single‐cell landscape, intercellular interactions, and tumor microenvironment in glioblastoma. The GSE154795 dataset contains 40 single‐cell RNA sequencing profiles from both newly diagnosed and recurrent glioblastoma cases. The GSE167960 dataset includes six freshly resected recurrent glioma samples (one WHO Grade IV glioma and five WHO Grade III gliomas) obtained immediately after resection during surgery. The GSE174554 dataset captures 80 human IDH wild‐type GBM samples through single‐nucleus RNA sequencing, evenly divided between primary tumors and recurrent tumors from the same patients. The cell type annotation in the GSE154795, GSE167960 and GSE174554 datasets was facilitated by reference mapping based on well annotated GBmap. This process utilized the “TransferData” function from the “Seurat” R package to migrate categorical or continuous data within a unified metadata framework.^[^ ^90^ ^]^

### Molecular Docking Analysis

Molecular docking was performed using AutoDock Vina (v1.1.2) to investigate ligand‐TRAF3IP3 interactions. The three‐dimensional structure of TRAF3‐interacting JNK‐activating modulator (TRAF3IP3; UniProt ID: Q9Y228) was retrieved from the AlphaFold Protein Structure Database^[^ ^91^ ^]^ (https://alphafold.ebi.ac.uk/). Chemical structures of sorafenib, crizotinib, and palbociclib were generated and energy‐minimized using Chem3D 21.0.0 with the MM2 force field. The TRAF3IP3 structure was then preprocessed by removing crystallographic water molecules and adding polar hydrogen atoms using Autodock Vina. Final protein and ligand structures were converted to PDBQT formats for grid parameterization.

The grid box centered at *x* = 18.20, *y* = 0.00, *z* = 14.32 (dimensions: 54 × 50 × 66 Å) to encompass potential binding pockets. The conformation with the most favorable binding free energy (ΔG) was selected for each ligand and visualized in PyMOL v2.2.0.

### Statistical Analysis

All data processing, statistical analysis, and visualization were performed using R 4.3.2 software. Correlations between two continuous variables were assessed using Spearman's correlation coefficient. The Wilcoxon rank‐sum test was applied to compare continuous variables among different groups. For the univariate cox regression and the multivariate cox regression, the “coxph” function was utilized from the survival package.^[^ ^92^ ^]^ The “ssGSEA” algorithm of the GSVA package^[^ ^82^ ^]^ was used to characterize the activity of pathways within tumor cells by calculating the enrichment of collected gene lists. All statistical tests were two‐sided and a *p*‐value of < 0.05 was considered statistically significant. The work has been reported in line with the REMARK criteria.^[^ ^93^ ^]^

### Availability of data and materials

1. **Transcriptomic Sequencing Data**: High‐throughput sequencing (RNA‐seq) of transcriptomic data was obtained from The Cancer Genome Atlas (TCGA, https://portal.gdc.cancer.gov, specifically TCGA‐LGG and TCGA‐GBM), Chinese Glioma Genome Atlas (CGGA, http://www.cgga.org.cn/, specifically CGGA693, CGGA325) and The Glioma Longitudinal AnalySiS (GLASS, https://glass‐consortium.org/), and microarray of transcriptomic data were obtained from Gene Expression Omnibus (GEO, http://www.ncbi.nlm.nih.gov/geo), ArrayExpress (https://www.ebi.ac.uk/arrayexpress/) and CGGA, including GSE108474 (Rembrandt cohort, https://www.ncbi.nlm.nih.gov/geo/query/acc.cgi?acc = GSE108474), GSE16011 (Gravendeel cohort, https://www.ncbi.nlm.nih.gov/geo/query/acc.cgi?acc = GSE16011), E‐MTAB‐3892 (Kamoun cohort, https://www.ebi.ac.uk/biostudies/arrayexpress/studies/E‐MTAB‐3892?accession = E‐MTAB‐3892) and CGGA301.
2. **Pan‐Cancer RNA‐seq and Immunotherapy Cohort Data**: Pan‐cancer RNA‐seq data and survival information were accessed from TCGA (33 types of cancer) by the “TCGAbiolinks” package.^[^ ^74^ ^]^ Pan‐cancer immunotherapy cohorts with survival information were accessed from Tumor Immunotherapy Gene Expression Resource portal (TIGER, http://tiger.canceromics.org/#/), including PRJNA482620^[^ ^94^ ^]^ (anti‐PD‐1. glioblastoma), GSE78220^[^ ^95^ ^]^ (anti‐PD‐1, melanoma), GSE91061^[^ ^96^ ^]^ (anti‐PD‐1, melanoma), Braun^[^ ^97^ ^]^ (anti‐PD‐1, Renal cell carcinoma), PRJEB23709^[^ ^98^ ^]^ (anti‐PD‐1+anti‐CTLA‐4, melanoma), Nathanson^[^ ^99^ ^]^ (anti‐CTLA‐4, melanoma). IMvigor210Core dataset (anti‐PD‐L1, muscle‐invasive urothelial carcinoma) was accessed by IMvigor210CoreBiologies package.^[^ ^100^ ^]^
3. **Drug Sensitivity Datasets**: Drug sensitivity datasets in Cancer v1 (GDSC1, pSet name: GDSC_2020 (v1‐8.2)), Drug Sensitivity in Cancer v2 (GDSC2, pSet name: GDSC_2020 (v2‐8.2)), CCLE (Cancer Cell Line Encyclopedia, pSet name: CCLE_2015), Cancer Therapeutics Response Portal v2(CTRP2, CTRPv2_2015) and Glioblastoma Multiforme (GBM, pSet name: GBM_2021_scr3) were downloaded via the “pharmacoGx” package.^[^ ^101^ ^]^
4. **Single‐Cell Transcriptomic Data**: Single‐cell transcriptomes of glioma samples from public datasets were downloaded from the GBmap, GSE154795 (https://www.ncbi.nlm.nih.gov/geo/query/acc.cgi?acc = GSE154795), GSE167960 (https://www.ncbi.nlm.nih.gov/geo/query/acc.cgi?acc = GSE167960) and GSE174554 (https://www.ncbi.nlm.nih.gov/geo/query/acc.cgi?acc = GSE174554). The data of Gusu in‐house cohort were available on Zenodo at https://zenodo.org/uploads/15165172.

## Conflict of Interest

The authors declare no conflict of interest.

## Author Contributions

Y. Y. and F. W. contributed equally to this work. F. W. and Y. Y. designed the study. F. W., Y. Y. and C. Z. contributed to bioinformatics analysis. F. W., H. D. and L. Z. conducted cellular experiments. F. W., K. H., R. H. and Y. L. wrote the manuscript. Y. Y. and Z. C. evaluated the immunohistochemical staining results. Y. Y., R. H., Y. L. and J. W. revised the manuscript. X. T. performed molecular docking analysis. D. L. and Y. Z. collected glioma tissues from surgery and corresponding clinical information. Z. C., Y. Yu and Z. W. involved in supervision and funding acquisition.

## Supporting information



Supporting Information

Supplemental Table 1

## Data Availability

The data that support the findings of this study are available from the corresponding author upon reasonable request.
