# Supplementary material for: Comprehensive Characterization of the Immune Microenvironment Based on Nested Resampling Machine Learning Framework Identifies TRAF3 Interacting Protein 3 as a Promising Regulator to Improve the Resistance to Immunotherapy in Glioma
Source: Adv Sci (Weinh). 2025 Jul 26;12(35):e02271. doi: 10.1002/advs.202502271 (PMC12462956; doi:10.1002/advs.202502271)
Supplement: Supplementary file 1 — Supporting Information [file ADVS-12-e02271-s001.pdf]

ADVANCED SCIENCE
Open Access

Supporting Information

for *Adv. Sci.*, DOI 10.1002/advs.202502271

# Comprehensive Characterization of the Immune Microenvironment Based on Nested Resampling Machine Learning Framework Identifies TRAF3 Interacting Protein 3 as a Promising Regulator to Improve the Resistance to Immunotherapy in Glioma

*Yanbo Yang, Fei Wang, Yulian Zhang, Run Huang, Chuanpeng Zhang, Lu Zhao, Hanhan Dang, Xinyu Tao, Yue Lu, Dengfeng Lu, Yunsheng Zhang, Kun He, Jiancong Weng, Zhouqing Chen*, Zhong Wang* and Yanbing Yu**

# Supplementary Figures

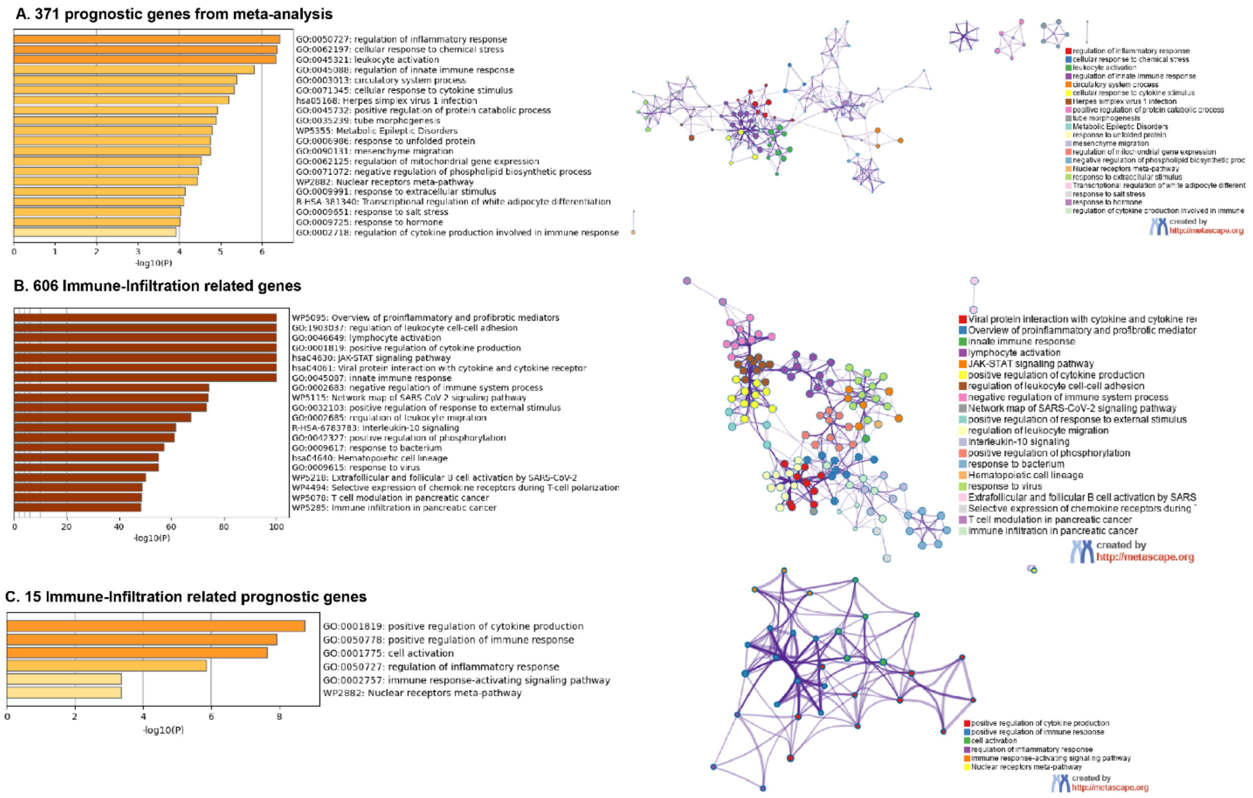


**Fig. S1: Enrichment analysis results from Metascape**

(A) Metascape analysis of 371 prognostic genes.

(B) Metascape analysis of 606 immune infiltration-related genes.

(C) Metascape analysis of 15 infiltration-related genes associated with glioma patient survival.

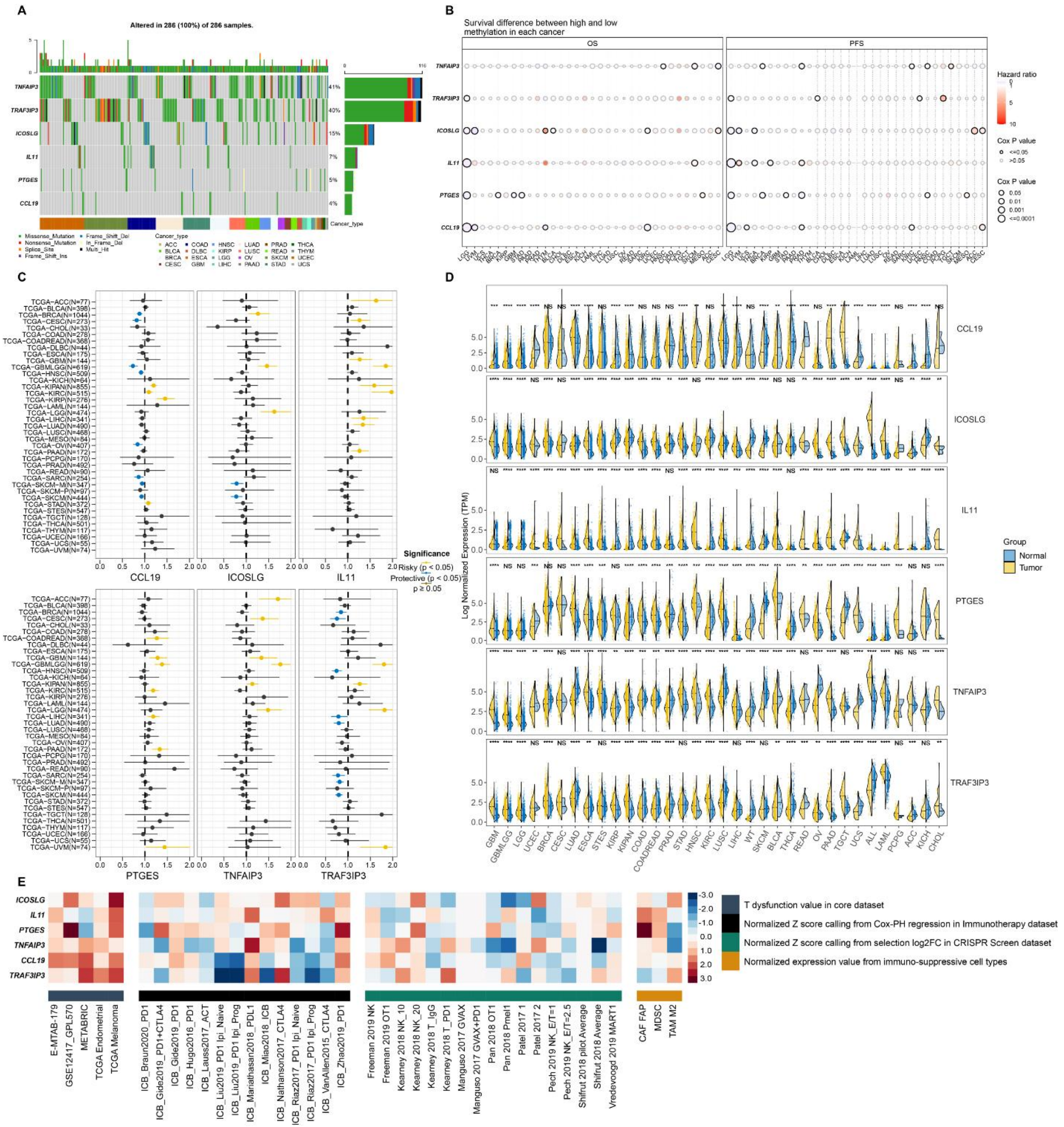


**Fig. S2: Multi-omics landscape of the IGLoS signature at the pan-cancer level**

(A) Genomic landscape of the IGLoS signature in pan-cancer. The waterfall plot presents 286 samples with at least one SNV.

(B) Epigenetic landscape of the IGLoS signature in pan-cancer. The bubble plot demonstrates the correlation between methylation levels of the IGLoS signature and their impact on overall survival.

(C, D) Transcriptomic landscape of the IGLoS signature in pan-cancer. The forest plot illustrates the association between gene expression levels of the IGLoS and their impact on overall survival in pan-cancer. The violin plot shows significant differences between tumor and normal tissues in pan-cancer.

(E) Immunogenomic landscape of the IGLoS signature in pan-cancer. The heatmap

from the “Gene set prioritization” module of the TIDE portal identifies ICOSLG as the optimal target to render the TME ICI-resistant. Genes (row) are ranked by their weighted average value across four immunosuppressive indices (columns), including the T cell dysfunction score, the T cell exclusion score, the association with ICB survival outcome, and the log-fold change (logFC) in CRISPR screens. The dysfunction score shows how a gene interacts with cytotoxic T cells to influence patient survival outcomes, whereas the T cell exclusion score assesses the gene expression levels in immunosuppressive cell types that drive T cell exclusion. The association score (z-score in the Cox-PH regression) of the ICB survival outcome evaluates genes whose activities are correlated with ICB benefit. The normalized logFC value in CRISPR screens facilitates the identification of regulators whose knockout can mediate the efficacy of lymphocyte-mediated tumor killing in cancer models.

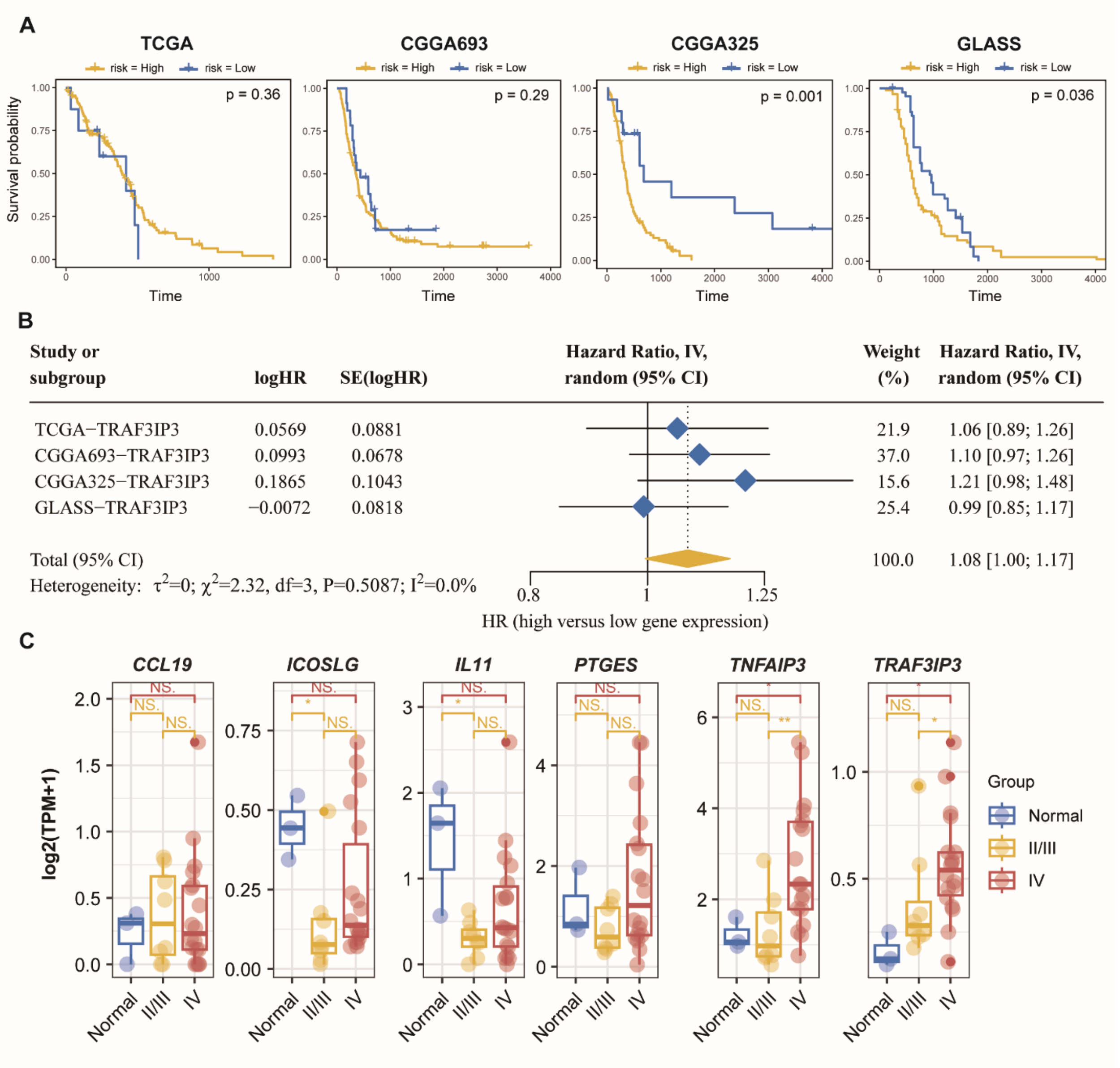


**Fig. S3: Prognostic validation of the IGLoS signature incorporating TRAF3IP3 in IDH wild-type GBM through survival analysis and in-house expression profiling**

(A) The Kaplan–Meier curve showing the possible ability of the IGLoS score to predict survival in four RNA-seq cohorts of IDH wild-type GBM.

(B) Forest plot showing the meta-analysis result of TRAF3IP3 in four RNA-seq cohorts of IDH wild-type GBM.

(C) Box plot showing the expression of IGLoS signature in the Gusu in-house dataset.

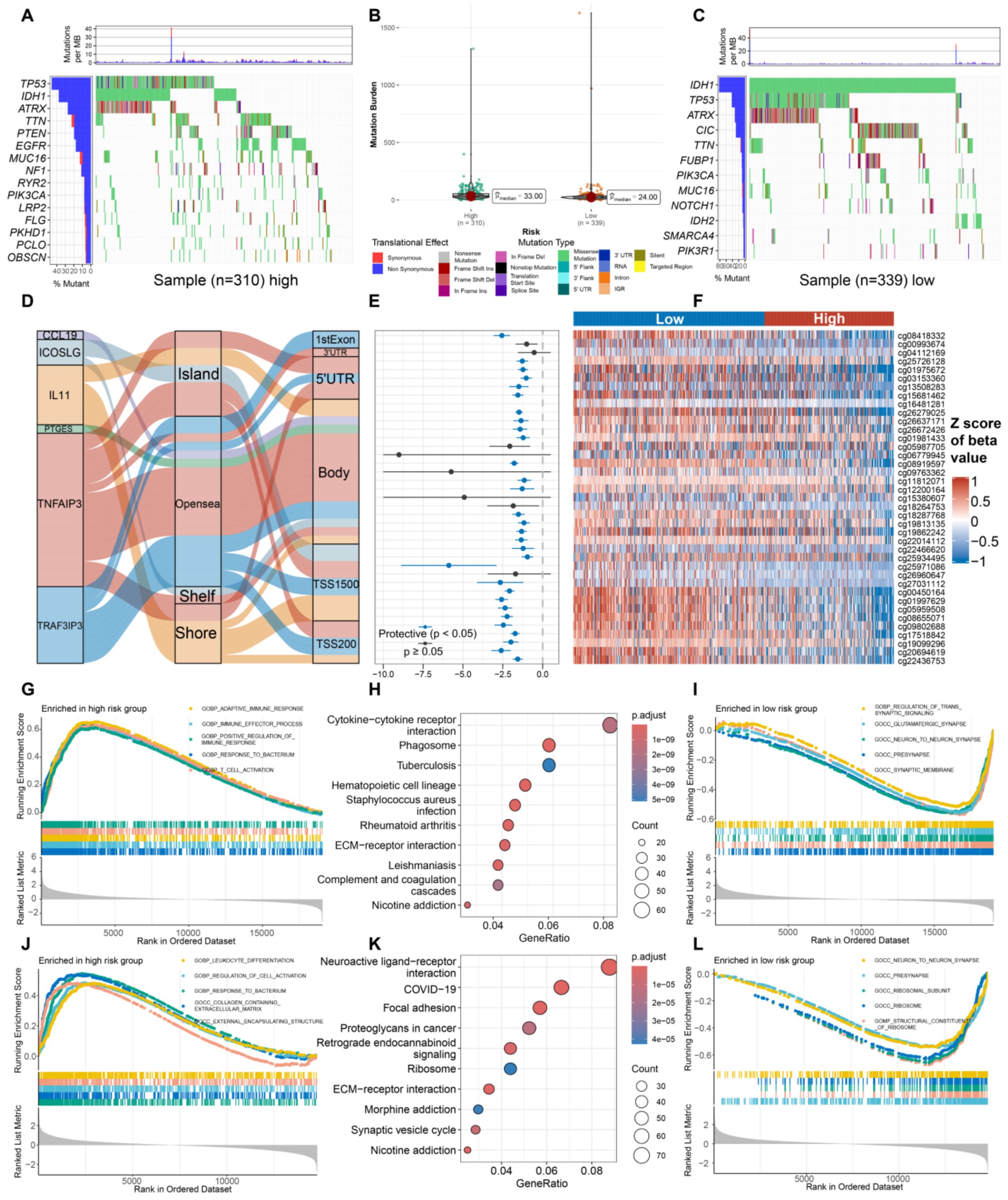

**Fig. S4: Genomic, Epigenetic, and Transcriptomic Analysis of the IGLoS in TCGA-LGG and TCGA-GBM**

(A, C) The waterfall plot displayed the top 10 mutated genes in the high-IGLoS and low-IGLoS group,

(B) The IGLoS score was positively correlated with the TMB.

(D) The annotation information of 39 differentially methylated sites.

(E) The univariate Cox regression analysis identifies 31 of 39 differentially methylated sites as protective factors.

(F) The heatmap shows a decreasing beta-value of methylation probes with increasing IGLoS score.

(G, I) GSEA of differentially expressed genes in TCGA.

(K) KEGG enrichment analysis of differentially expressed genes in TCGA.

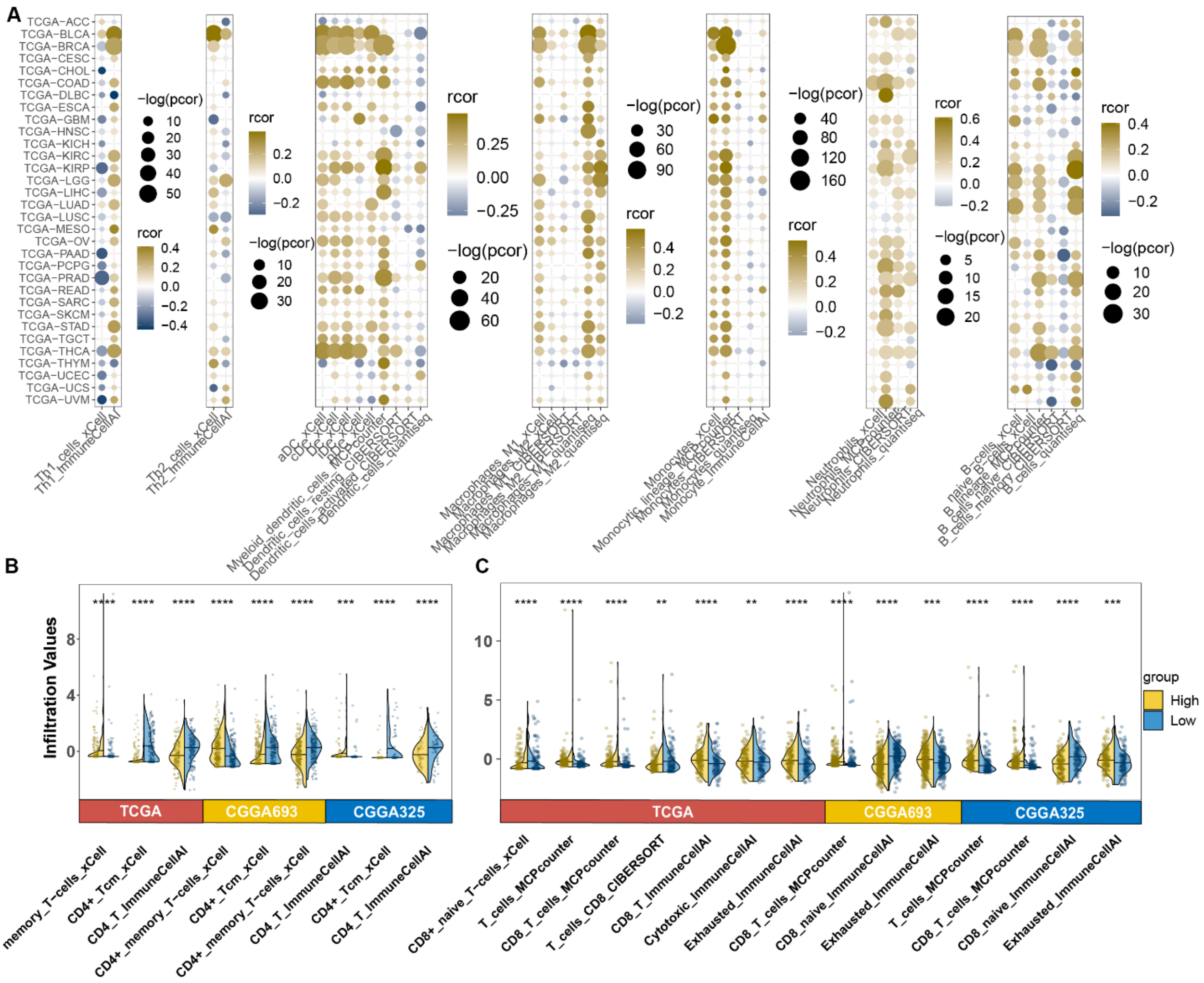


**Fig. S5: The IGLoS score depicts the activity of the TME in TCGA pan-cancer cohorts and four RNA-seq cohorts of glioma**

(A) Correlation between IGLoS scores and immune cell infiltration at the pan-cancer level.

(B) Comparison of CD4+ T-cell infiltration between the high-IGLoS and low-IGLoS groups in patients with glioma.

(C) Comparison of CD8+ T-cell infiltration between the high-IGLoS and low-IGLoS groups in patients with glioma.

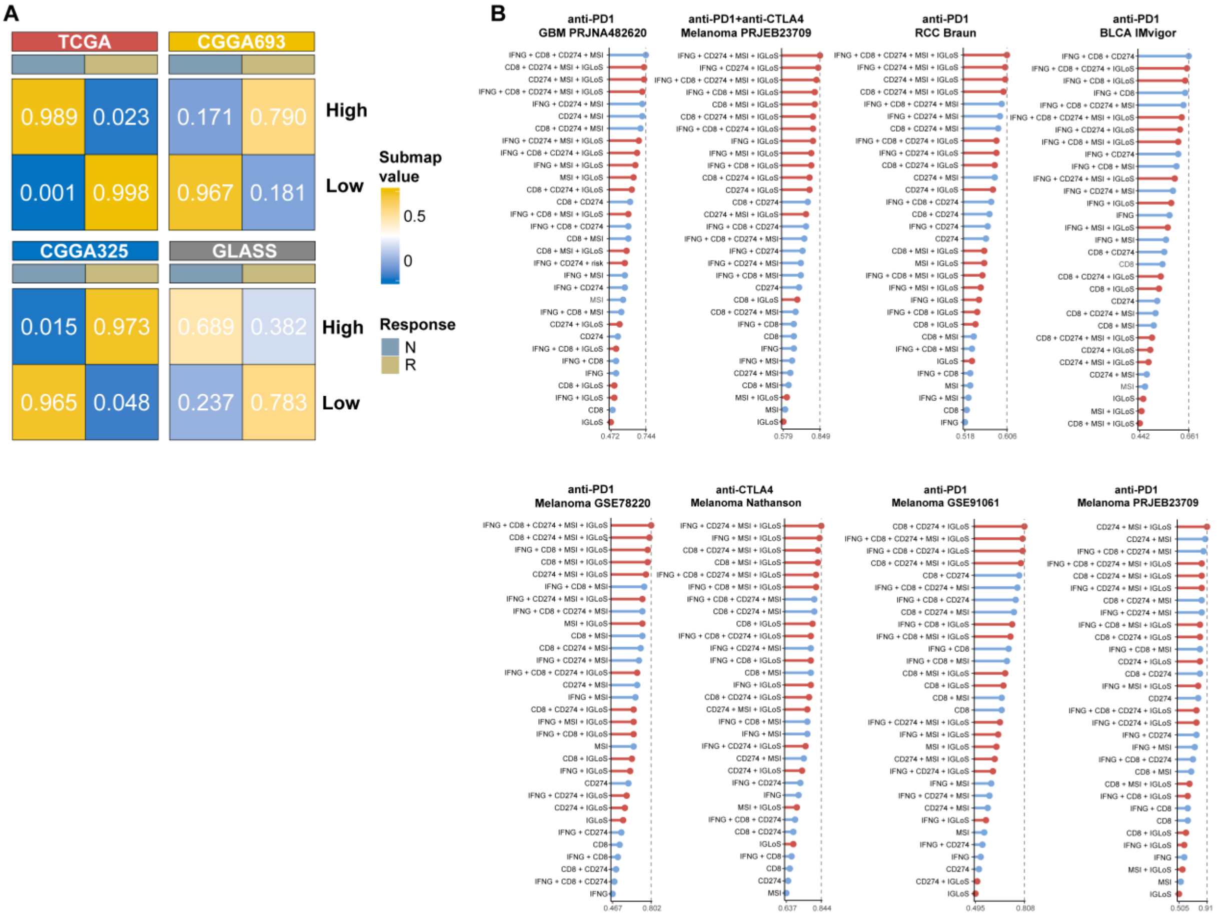


**Fig. S6: Prediction of response to ICI and comparison of combinatorial predictors of response to ICI**

(A)The subclass mapping algorithm shows the consistency between the prediction of response to ICI and the IGLoS groups.

(B) Comparison of individual predictors and combinatorial predictors in PRJNA482620 (glioblastoma), PRJEB23709 (Melanoma), Braun (RCC), IMvigor (BLCA), GSE78220 (Melanoma), Nathanson (Melanoma), GSE91061 (Melanoma), and PRJEB23709 (Melanoma).

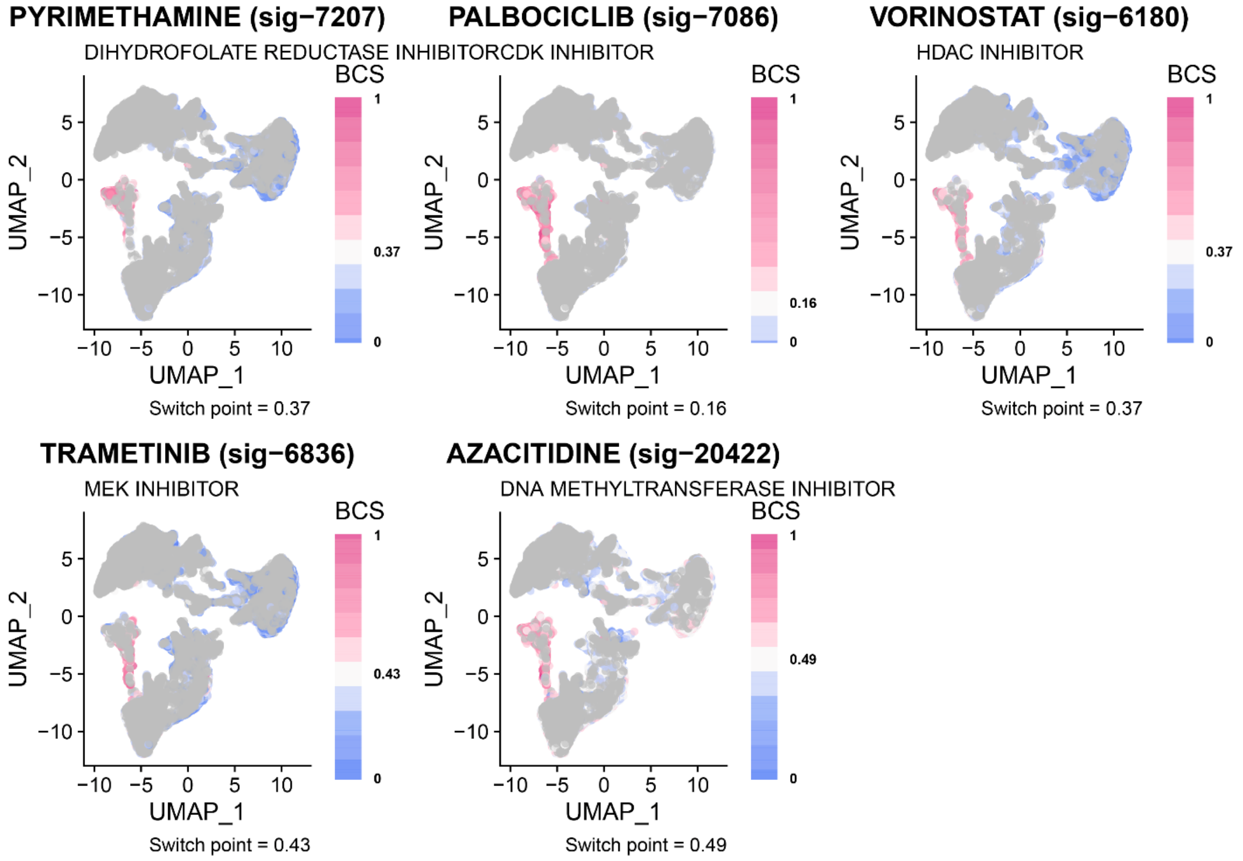


**Fig. S7: Beyond-cell scores of the other five drug candidates in at least two pharmacogenomic databases**

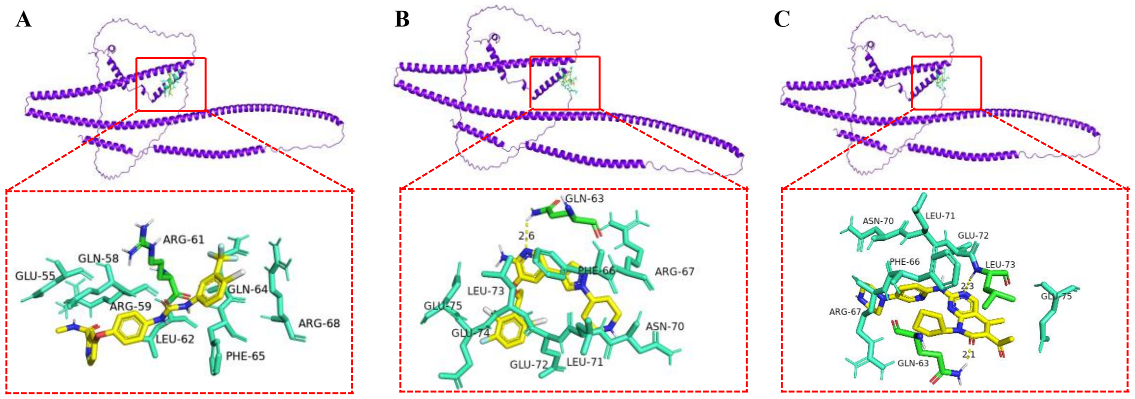


**Fig. S8 Molecular recognition mechanisms of TRAF3IP3-ligand complexes.**

(A) Binding pose of sorafenib within the hydrophobic pocket of TRAF3IP3. The hydrogen bond with the backbone carbonyl oxygen of Arg61 is shown as yellow dashed lines (2.4 Å). Van der Waals interactions involve Glu55, Gln58, Arg59, Leu62, Gln64, Phe65 and Arg68 (ΔG = -6.5 kcal/mol).

(B) Binding pose of crizotinib within the hydrophobic pocket of TRAF3IP3. The hydrogen bond with the backbone amide hydrogen of Gln63 (2.6 Å) is shown as yellow dashed lines (2.6 Å). Van der Waals interactions involve Phe66, Arg67, Leu71, Glu72, Leu73, Glu74 and Glu75 (ΔG = -6.7 kcal/mol).

(C) Binding pose of palbociclib within the hydrophobic pocket of TRAF3IP3. Two hydrogen bonds are shown as yellow dashed lines: (i) carboxyl oxygen with Gln63 (2.1 Å), and (ii) pyrimidine nitrogen with Leu73 (2.3 Å). Van der Waals interactions include Arg67, Asn70, Leu71, Glu72 and Glu75 (ΔG = -6.9 kcal/mol).

Molecular docking was performed using AutoDock Vina v1.1.2 (exhaustiveness = 8, grid center: x= 18.20, y= 0.00, z= 14.32; grid dimensions: 54 × 50 × 66 Å). Binding free energy (ΔG) values are indicated in parentheses. All structures were visualized in PyMOL (v2.2.0).

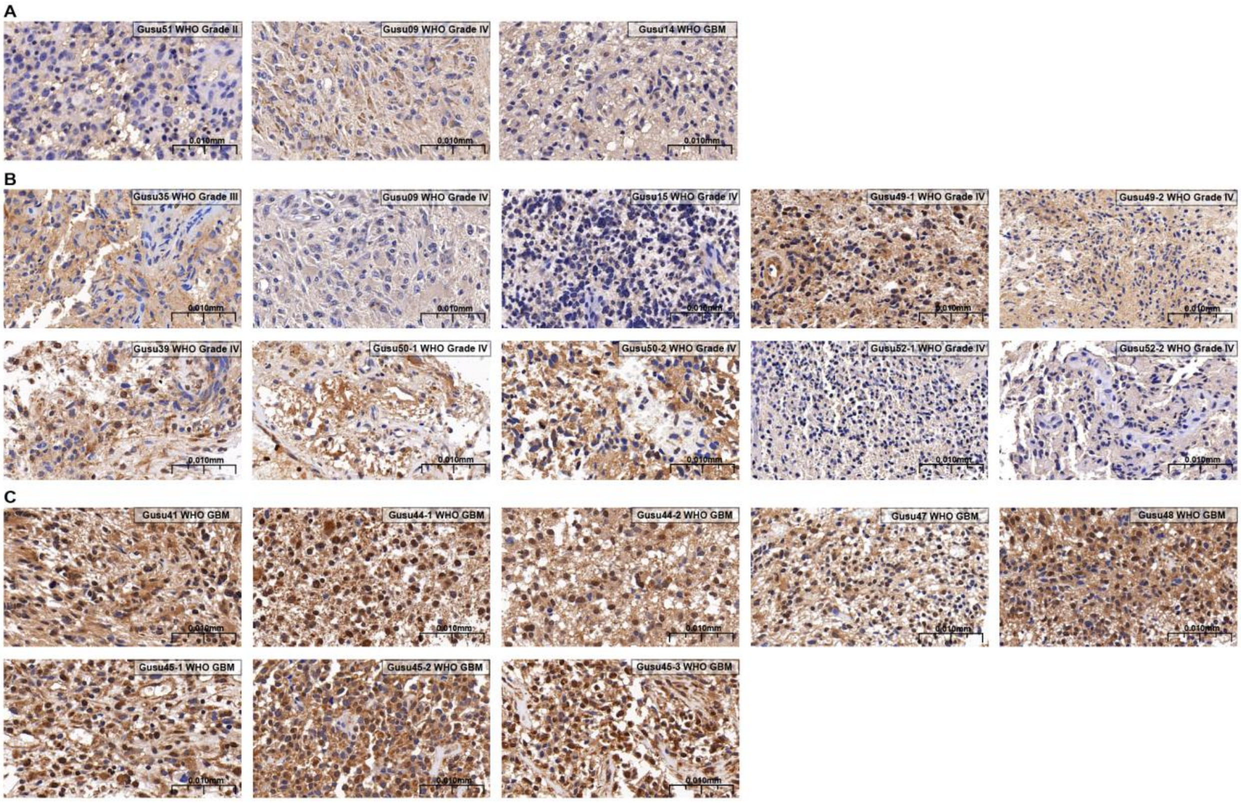


**Fig. S9: Representative IHC staining images of TRAF3IP3 and PDL1 from in-house glioma cohort.**

(A) Representative IHC staining images of PDL1 for glioma patients of WHO grade II (left panel), IV (middle panel) and GBM (right panel).

(B) Representative IHC staining images of TRAF3IP3 for glioma patients of WHO grade II to IV and GBM.

(B) Representative IHC staining images of TRAF3IP3 for glioma patients of WHO GBM.

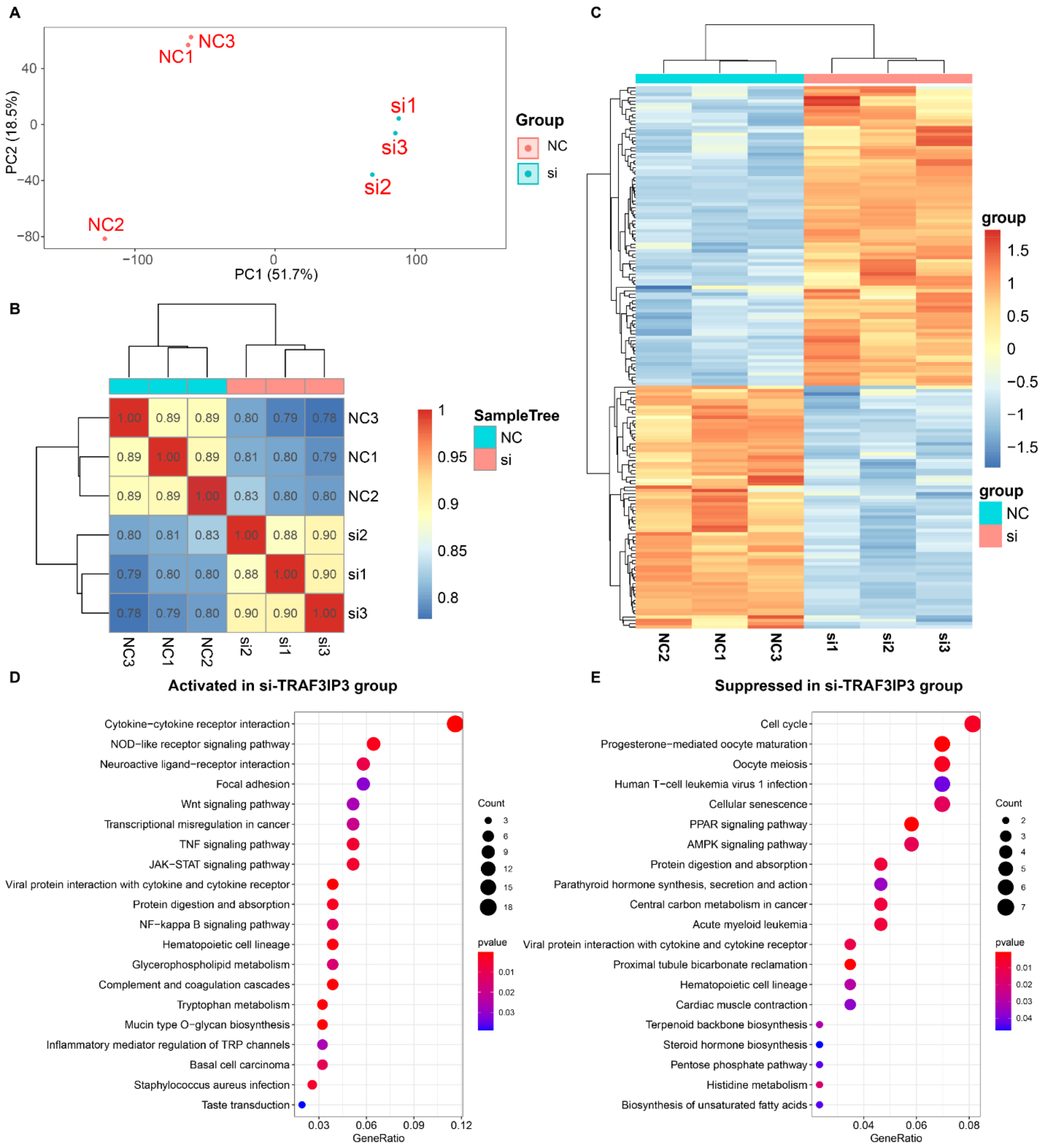


**Fig. S10: Differential expression analysis and KEGG enrichment analysis between TRAF3IP3-knockdown cells and cells in the control group**

(A) PCA analysis between TRAF3IP3-knockdown cells and cells in the control group.

(B) Inter-sample correlation analysis between TRAF3IP3-knockdown cells and cells in the control group.

(C) Heatmap showing results of differential expression between TRAF3IP3-knockdown cells and cells in the control group.

(D–E) KEGG analysis showing activated pathways (D) and suppressed pathways (E) after TRAF3IP3 knockdown.

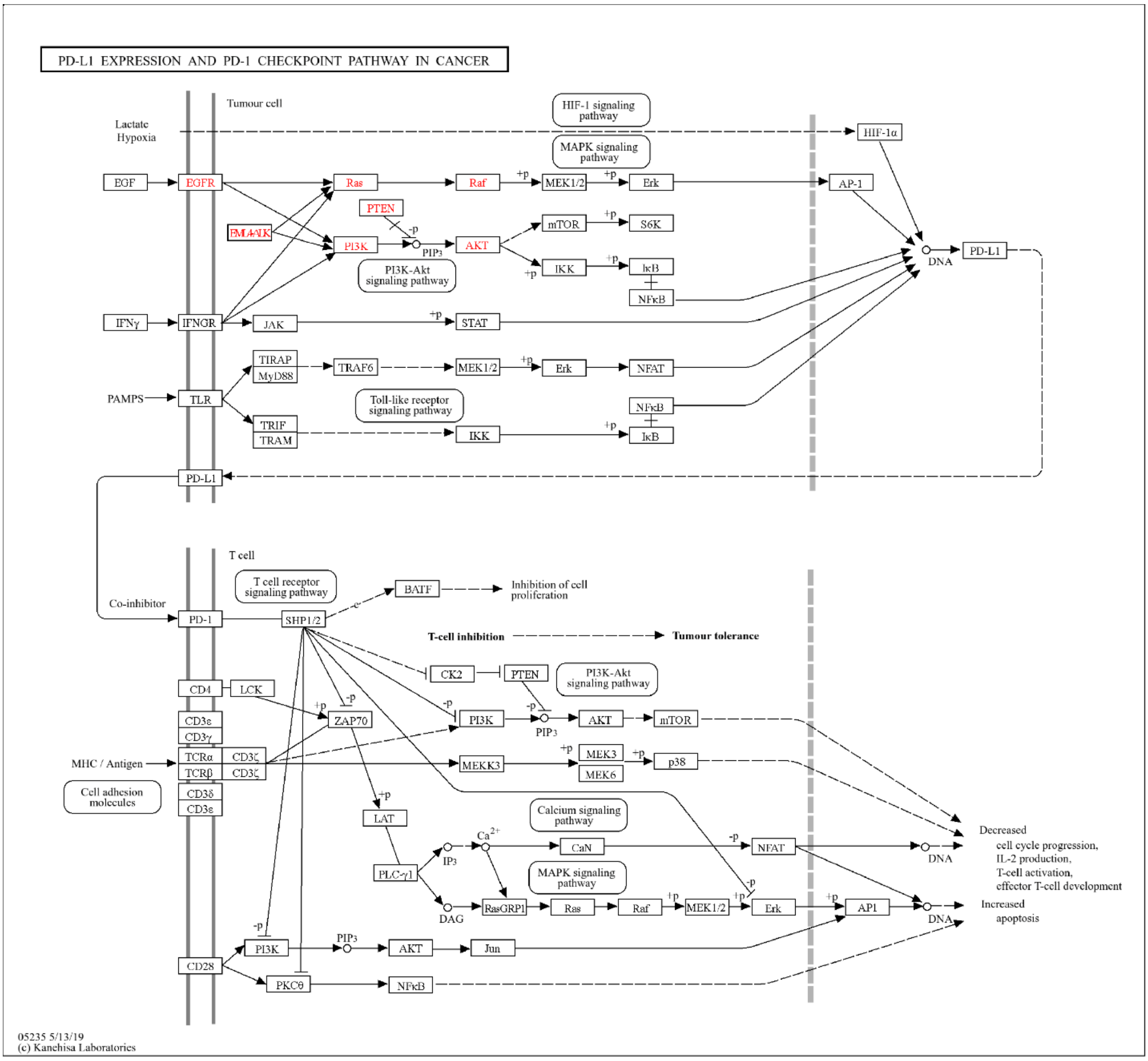


**Fig. S11 KEGG pathway map of PD-L1 expression and PD-1 checkpoint pathway in cancer (map05235).**

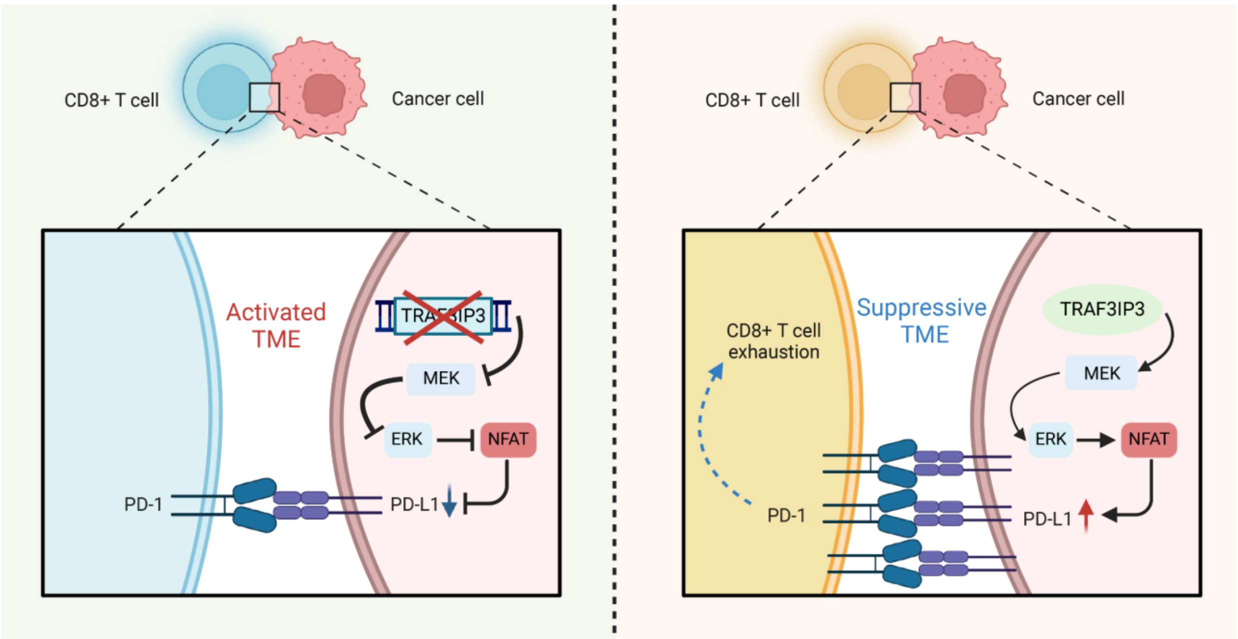


**Fig. S12 TRAF3IP3 in glioma cells upregulates PDL1 expression through transcriptional activation of PDL1 by NFATC2 via the ERK pathway, leading to exhaustion of CD8+ T cells in the TME, which results in the overall suppression of the TME, and thus resistance to immunotherapy.**

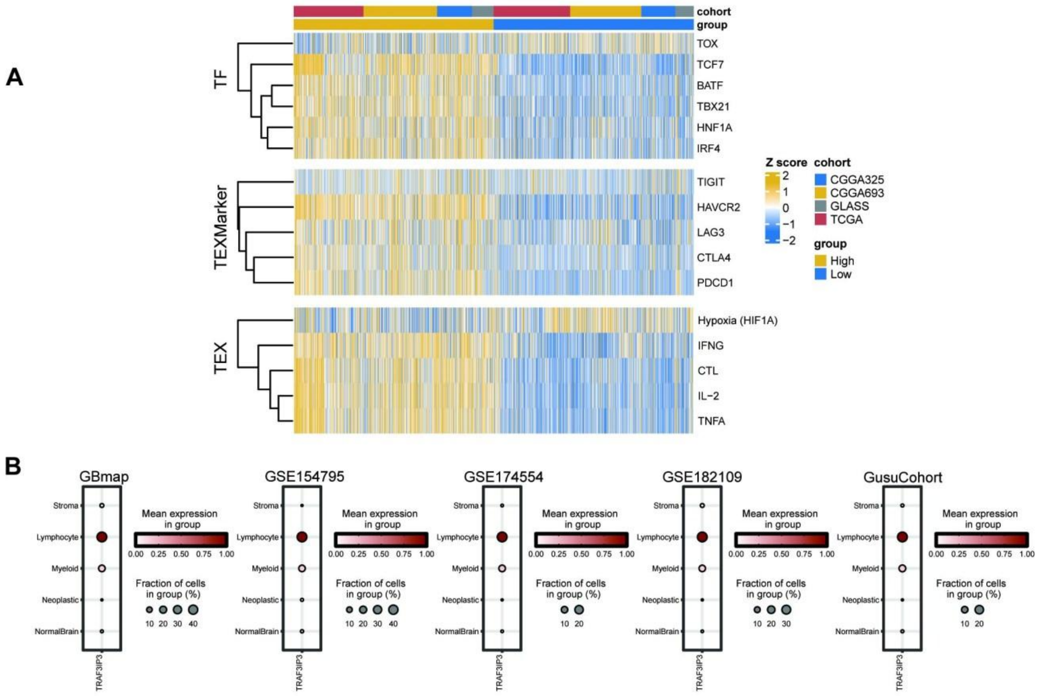


**Fig. S13 The association between TRAF3IP3 and T-cell exhaustion at bulk RNA sequencing resolution and single-cell resolution.**

(A) Heatmap showing the expression of a series of regulators representing the degree of T cell exhaustion between the high-expression group and the low-expression group of TRAF3IP3.

(B) Bubble plot showing the mean expression of TRAF3IP3 in five dependent cohorts at the single-cell level.

## Supplementary Tables

**Supplementary Table 1** Source of cohorts used in this study

**Supplementary Table 2** Gene list of 17 immune cell signatures and 15 immune function signatures from comprehensive literature exploration

**Supplementary Table 3** Signatures Related to Intrinsic Pathways Causing Resistance to ICI

**Supplementary Table 4** siRNA sequences used in this study

**Supplementary Table 5** Clinical features of patients from the Gusu in-house dataset.

**Supplementary Table 6** Prognostic genes from the meta-analysis of four RNA-seq cohorts

**Supplementary Table 7** Prognostic genes from the meta-analysis of four microarray cohorts

**Supplementary Table 8** The IGLoS scores in four RNA-seq cohorts of glioma, TCGA pan-cancer cohorts, and immunotherapy cohorts using the gradient boosting model

**Supplementary Table 9** Clinical characteristics of different IGLoS subgroups in four RNA-seq cohorts

**Supplementary Table 10** Differentially Methylated Sites between High and Low IGLoS Groups in TCGA-LGG and TCGA-GBM

**Supplementary Table 11** Potential drugs through the Wilcoxon rank-sum test
